# Supplementary material for: Evaluation of continuous arrhythmia monitoring using an implantable loop recorder in heart failure patients with a reduced ejection fraction: The LINQ2-HF trial rationale and protocol
Source: PLoS One. 2025 Aug 28;20(8):e0321604. doi: 10.1371/journal.pone.0321604 (PMC12393711; doi:10.1371/journal.pone.0321604)
Supplement: S2 File — (DOCX) [file pone.0321604.s002.docx]

**S2. Copy of the protocol that was approved by the ethics committee (Japanese)**

左室駆出率の低下した心不全症例における植込み型ループレコーダーを用いた不整脈連続モニタリングの有用性の検討

臨床研究計画書

（特定臨床研究）

研究計画書番号： NR2024-003

　　 2024年7月13日　　第1版

**略語一覧**

| 略語 | 説明 | |
| --- | --- | --- |
| AF | Atrial fibrillation | 心房細動 |
| BNP | Brain natriuretic peptide | 脳性ナトリウム利尿ペプチド |
| CIED | Cardiovascular implantable electronic device | 植込み型心臓デバイス |
| CRB | Certified Review Board | 臨床研究審査委員会 |
| CRF | Case Report Form | 症例報告書 |
| CTCAE | Common Terminology Criteria for Adverse Events | 有害事象 共通用語規準 |
| EDC | Electric Data Capture | 電子的臨床検査情報収集 |
| FAS | Full analysis set | 最大の解析対象集団 |
| GCP | Good Clinical Practice | 医薬品の臨床試験の実施の基準に関する省令 |
| HFrEF | Heart failure with reduced ejection fraction | 左室駆出率の低下した心不全 |
| ICD | Implantable cardioverter defibrillator | 植込み型除細動器 |
| ILR | Implantable loop recorder | 植込み型ループレコーダー |
| IVS | Interventricular septum thickness | 心室中隔厚 |
| jRCT | Japan Registry of Clinical Trials | 臨床研究等提出・公開システム |
| LAD | Left atrial diameter | 左房径 |
| LVDd | Left ventricular end-diastolic diameter | 左室拡張末期径 |
| LVDs | Left ventricular end-systolic diameter | 左室収縮末期径 |
| LVEF | Left ventricular ejection fraction | 左室駆出率 |
| LVPW | Left ventricle posterior wall thickness | 左室後壁厚 |
| PMDA | Pharmaceuticals and Medical Devices Agency | 医薬品医療機器総合機構 |
| PPS | Per protocol set | 研究計画書に適合した対象集団 |
| SAS | Safety Analysis Set | 安全性解析対象集団 |
| SGLT | Sodium glucose co-transporter | ナトリウム－グルコース共輸送体 |
| TIA | Transient ischemic attack | 一過性脳虚血発作 |

**目　次**

[１．概要 6](#_Toc169625035)

[２．臨床研究の実施体制 9](#_Toc169625036)

[３．臨床研究の背景 9](#_Toc169625037)

[４．臨床研究の目的 11](#_Toc169625038)

[５．臨床研究の内容に関する事項 11](#_Toc169625039)

[5.1　主要評価項目および副次評価項目 11](#_Toc169625040)

[5.2　臨床研究のデザインおよびアウトライン 12](#_Toc169625041)

[5.2.1　臨床研究のデザイン 12](#_Toc169625042)

[5.2.2　臨床研究のアウトライン 12](#_Toc169625043)

[5.3　症例登録・割付方法 13](#_Toc169625044)

[5.4　研究機器の概要 13](#_Toc169625045)

[5.5　研究機器の管理の手順 16](#_Toc169625046)

[5.6　研究対象者の参加予定期間 16](#_Toc169625047)

[5.7　臨床研究全体の中止基準 16](#_Toc169625048)

[６．研究対象者の選択および除外関する基準 16](#_Toc169625049)

[6.1　研究対象者（対象疾患） 16](#_Toc169625050)

[6.2　選択基準 16](#_Toc169625051)

[6.3　除外基準 17](#_Toc169625052)

[７．研究対象者に対する治療に関する事項 18](#_Toc169625053)

[7.1　研究治療の実施手順 18](#_Toc169625054)

[7.1.1　同意取得～登録、登録時評価 18](#_Toc169625055)

[7.1.2　ILRの植込み 18](#_Toc169625056)

[7.1.3　遠隔モニタリング 20](#_Toc169625057)

[7.1.4　外来フォローアップ 21](#_Toc169625058)

[7.1.5　研究の終了 21](#_Toc169625059)

[7.2　観察・検査項目およびスケジュール 22](#_Toc169625060)

[7.2.1　スケジュール 22](#_Toc169625061)

[7.2.2　観察・検査項目 22](#_Toc169625062)

[7.3　併用薬（療法）に関する規定 25](#_Toc169625063)

[7.3.1　併用可能・制限薬（療法） 25](#_Toc169625064)

[7.3.2　併用禁止薬（療法） 25](#_Toc169625065)

[7.4　研究対象者への指導事項 25](#_Toc169625066)

[7.5　研究終了後の対応 26](#_Toc169625067)

[7.6　研究対象者ごとの中止基準 26](#_Toc169625068)

[８．有効性の評価に関する事項 27](#_Toc169625069)

[8.1　有効性の評価指標 27](#_Toc169625070)

[8.1.1　主要評価項目（Primary endpoint） 27](#_Toc169625071)

[8.1.2　副次評価項目（Secondary endpoint） 27](#_Toc169625072)

[8.2　有効性評価指標に関する評価、記録 27](#_Toc169625073)

[8.3　有効性評価指標に関する解析の方法ならびに時期 28](#_Toc169625074)

[９．安全性の評価に関する事項 28](#_Toc169625075)

[9.1　安全性の評価指標 28](#_Toc169625076)

[9.2　安全性評価指標に関する評価、記録、解析の方法ならびに実施時期 28](#_Toc169625077)

[9.3　安全性評価指標に関する解析の方法ならびに実施時期 29](#_Toc169625078)

[9.4　疾病等の情報収集、記録および報告に関する手順 29](#_Toc169625079)

[9.4.1　疾病等に関する定義 29](#_Toc169625080)

[9.4.2　疾病等報告の対象および報告期間 30](#_Toc169625081)

[9.4.3　疾病等報告に関する手順 31](#_Toc169625082)

[9.5　疾病等発生後の研究対象者の観察 32](#_Toc169625083)

[10．統計的な解析に関する事項 32](#_Toc169625084)

[10.1　解析対象集団 32](#_Toc169625085)

[10.1.1　最大の解析対象集団 (Full Analysis Set, FAS) 32](#_Toc169625086)

[10.1.2　研究計画書に適合した対象集団 (Per Protocol Set, PPS) 32](#_Toc169625087)

[10.1.3　安全性解析対象集団 (Safety Analysis Set, SAS) 32](#_Toc169625088)

[10.2　目標登録数と設定根拠 32](#_Toc169625089)

[10.3　症例の取扱い 33](#_Toc169625090)

[10.4　データの取扱い 33](#_Toc169625091)

[10.5　統計解析項目および解析計画 33](#_Toc169625092)

[10.5.1　研究対象者の背景の要約 33](#_Toc169625093)

[10.5.2　主要評価項目の解析 33](#_Toc169625094)

[10.5.3　副次評価項目の解析 33](#_Toc169625095)

[10.5.4　安全性評価項目の解析 34](#_Toc169625096)

[10.5.5　中間解析 34](#_Toc169625097)

[10.6　最終解析 35](#_Toc169625098)

[11．原資料等の閲覧に関する事項 35](#_Toc169625099)

[12．品質管理および品質保証に関する事項 35](#_Toc169625100)

[12.1　モニタリング 35](#_Toc169625101)

[12.2　監査 35](#_Toc169625102)

[13．倫理的な配慮に関する事項 36](#_Toc169625103)

[13.1　法令等の遵守 36](#_Toc169625104)

[13.2　予期される利益、負担および不利益 36](#_Toc169625105)

[13.3　研究対象者に係わる遺伝的特徴等に関する研究結果や偶発的初見の取扱い 36](#_Toc169625106)

[14．記録（データを含む）・試料の取扱いおよび保存に関する事項 37](#_Toc169625107)

[14.1　データ収集の方法 37](#_Toc169625108)

[14.2　記録の保存 37](#_Toc169625109)

[14.3　試料の保管等 38](#_Toc169625110)

[14.4　情報の保管と廃棄 38](#_Toc169625111)

[15．金銭の支払いおよび補償に関する事項 38](#_Toc169625112)

[15.1　金銭の支払い（研究対象者の費用負担） 38](#_Toc169625113)

[15.2　補償に関する事項 38](#_Toc169625114)

[16．情報の公表 38](#_Toc169625115)

[17．実施期間 39](#_Toc169625116)

[18．研究対象者に対する説明および同意 39](#_Toc169625117)

[18.1　説明文書および同意文書の作成 39](#_Toc169625118)

[18.2　同意取得（インフォームド・コンセント） 40](#_Toc169625119)

[19．利益相反に関する事項 40](#_Toc169625120)

[20．知的財産権 40](#_Toc169625121)

[21．個人情報等の取扱い 41](#_Toc169625122)

[21.1　個人情報の保護 41](#_Toc169625123)

[21.2　データの二次利用 41](#_Toc169625124)

[22．研究計画書の遵守および研究計画書の変更 41](#_Toc169625125)

[22.1　研究計画書の遵守 41](#_Toc169625126)

[22.2　研究計画書の変更 41](#_Toc169625127)

[23．不適合の管理 41](#_Toc169625128)

[24．定期報告 42](#_Toc169625129)

[25．研究の中止 42](#_Toc169625130)

[26．研究の終了 42](#_Toc169625131)

[26.1　総括報告書の作成 42](#_Toc169625132)

[26.2　総括報告書の届出 43](#_Toc169625133)

**１．概要**

| **研究課題名** | 左室駆出率の低下した心不全症例における植込み型ループレコーダーを用いた不整脈連続モニタリングの有用性の検討 |
| --- | --- |
| **研究の目的** | 心房細動（AF: atrial fibrillation）と診断されていない、左室駆出率の低下した心不全（HFrEF: heart failure with reduced ejection fraction）患者に合併する無症候性不整脈の検出における、植込み型ループレコーダー（ILR: implantable loop recorder）を用いた持続心拍モニタリングの臨床的有用性を検討する。 |
| **研究デザイン** | 単施設、非盲検非対照研究 |
| **研究の性質** | 探索的研究 |
| **研究医療機器** | 一般的名称：　植込み型心電用データレコーダ  販売名：　　　　メドトロニック　LINQⅡ  分類：　　　　 　高度管理医療機器（クラスⅣ）  製造販売業者：日本メドトロニック株式会社  ※構成品として、「MyCareLink Relay Home Communicator」を含む。  一般的名称：　植込み能動型機器管理用プログラム  販売名：　　　　メドトロニック　Reveal LINQ モバイルマネージャ  分類：　　　　 　高度管理医療機器（クラスⅢ）  製造販売業者：日本メドトロニック株式会社  一般的名称：　植込み能動型機器用プログラマ  販売名：　　　　メドトロニック　24967 ペイシェントコネクタ  分類：　　　　 　高度管理医療機器（クラスⅢ）  製造販売業者：日本メドトロニック株式会社 |
| **対象** | AFと診断されていないHFrEF患者 |
| **選択基準** | 以下の基準のすべてに該当する患者を、本臨床研究に組み入れる。   1. HFrEF（左室駆出率40%以下）の患者 2. HFrEFに対して適切な薬物療法が行われ、外来通院中または外来通院が可能な患者 3. CHADS_2_スコアが1点以上の患者 4. 同意取得時の年齢が20歳以上の患者 5. 本臨床研究の参加に関して患者本人から文書で同意の得られた患者 |
| **除外基準** | 以下の基準のいずれかに該当する患者は、本臨床研究に組み入れない。   1. 上室性不整脈を検出可能な植込み型心臓デバイス（CIED: cardiovascular implantable electronic device。恒久的ペースメーカー、植込み型除細動器、ILRを指す）が植込まれている患者 2. 同意取得時にAFと診断されている患者 3. 生命予後1年以内と考えられる患者 4. 免疫不全状態にある患者 5. 活動性の感染症がある患者 6. 植込み部位の皮下組織が薄く、安全にILRを植込むことが困難と判断される患者 7. その他、研究責任（分担）医師が本研究への参加が不適当であると判断した患者 |
| **評価項目** | ※主たる解析は植込み後1年時のデータを用いて実施する。すべての症例が植込み後3年時の評価を完了したのちに最終解析を実施する。  **＜主要評価項目＞**  AFおよび非持続性または持続性の心室頻拍を認めた患者の割合  解析対象集団において、以下が認められた患者の割合と定義する。   - AF：6分間以上持続するもの - 非持続性心室頻拍：心拍数150回/分を超える頻拍で、16連発以上かつ持続時間30秒以下のもの - 持続性心室頻拍：心拍数150回/分を超える頻拍で、30秒以上持続するもの   **＜副次評価項目＞**   1. 4.5秒以上のポーズを認めた患者の割合 2. 心拍数30回/分以下の徐脈を認めた患者の割合 3. 高度房室ブロックを認めた患者の割合 4. 恒久的ペースメーカー、植込み型除細動器、カテーテルアブレーション、抗不整脈薬による治療介入が行われた患者の割合 5. 全死亡割合 6. 心血管死亡割合 7. 心血管疾患または心不全による入院を要した患者の割合 8. 脳卒中を発症した患者の割合   **＜安全性の評価項目＞**   1. ILR植込みと関連のある有害事象の発生割合 2. 不具合の発生割合 |
| **プロトコール治療** | 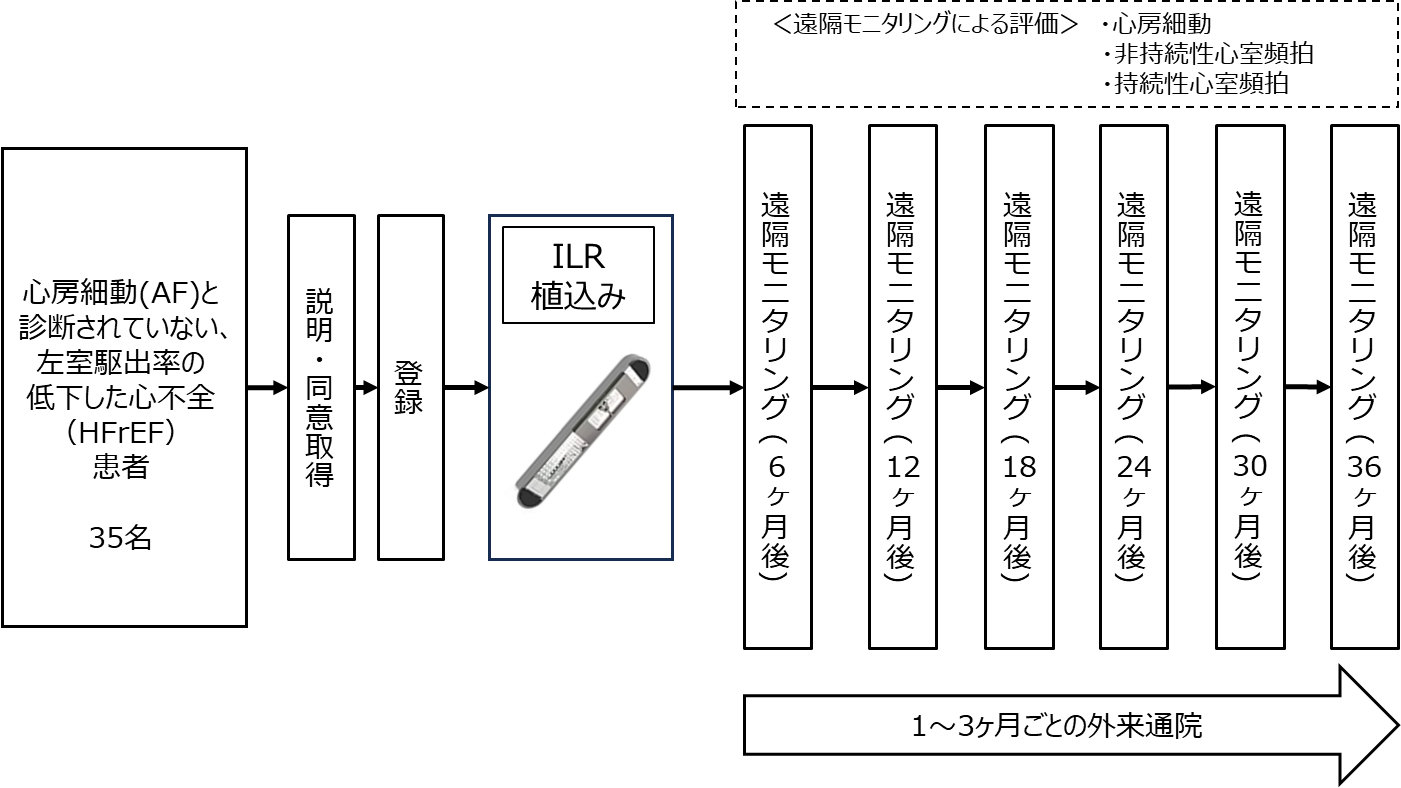  研究責任（分担）医師は、文書同意取得後に、局所麻酔科に左前胸部皮下にILRの植込みを行う。植込み後、6ヶ月ごとに遠隔モニタリング（メドトロニックCareLinkシステム）で、本研究の定める不整脈イベント（5.1章参照）を評価する。  主要評価項目および副次評価項目①、②、③に関しては、遠隔モニタリングにより発生が検出された場合は、外来担当医師とは別の評価担当医師にメールでアラートが送付又は転送される。評価担当当該医師は、記録された心電図をサーバ上で確認して、診断する（不整脈イベントの発生の有無を判定する）。評価担当医師が、医学的に早急に外来担当医師に伝えるべき内容と判断した場合は、随時、外来担当医師に遠隔モニタリングの所見を報告する。なお、患者に不整脈を疑う症状があった場合は、随時ILRのデータを確認する。  また、ILR植込み前と同様に、1～3ヶ月ごとの外来通院を行う。その中で、副次評価項目④～⑧に該当するイベントの有無等の研究対象者の状態を含む臨床経過を確認する。 |
| **目標研究対象者数** | 35名 |
| **研究実施期間** | 総研究期間：実施計画のjRCT公表日～2030年9月30日（6年）  （予定登録期間：2年、観察期間：登録終了後3年、解析期間：1年） |
| **研究施設数** | 単施設（東京医科歯科大学病院） |

**２．臨床研究の実施体制**

| 役割 | 名称 | 職名 | 所属機関・部署等※ |
| --- | --- | --- | --- |
| 研究責任医師 | 宮﨑　晋介 | 寄附講座准教授 | 東京医科歯科大学病院  循環器内科 |
| データマネジメント責任者 | 村上 妙子 | URA | 東京医科歯科大学 ヘルスサイエンスR&Dセンター |
| モニタリング責任者 | 谷口 順子 | URA | 東京医科歯科大学 ヘルスサイエンスR&Dセンター |
| モニタリング責任者 | 桑本 幸接 | URA | 東京医科歯科大学 ヘルスサイエンスR&Dセンター |
| 統計解析責任者 | 花澤 遼一 | 特任助教 | 東京医科歯科大学 ヘルスサイエンスR&Dセンター/臨床統計学分野 |
| 統計解析担当者 | 平川 晃弘 | 教授 | 東京医科歯科大学 臨床統計学分野 |
| 統計解析担当者 | 佐藤 宏征 | 助教 | 東京医科歯科大学 臨床統計学分野 |
| 統計解析担当者 | 北林 遼 | 特任助教 | 東京医科歯科大学 ヘルスサイエンスR&Dセンター/臨床統計学分野 |
| 研究開発計画支援  担当者 | 酒井 絢子 | URA | 東京医科歯科大学 ヘルスサイエンスR&Dセンター |
|  | 石黒　めぐみ | 准教授 |  |
| 遠隔モニタリング評価担当医師 | 立石 遼 | 助教 | 東京医科歯科大学病院  循環器内科 |
|  | 山尾 一哉 | 助教 |  |

※所在地：東京都文京区湯島1-5-45

**３．臨床研究の背景**

本邦における死因別死亡総数において、心疾患は2番目であり、心不全はその内訳で最も多い。社会の高齢化と共に心不全患者は増加の一途を辿っており、2020年には120万人に達するとされる^1^。そのうち半数は左室駆出率が低下した心不全であり、「急性･慢性心不全診療ガイドライン」（2017年改訂版）では、左室駆出率40%未満がHFrEFと定義されている^2^。

AFは心不全に最も多く合併する不整脈であり、重症心不全例ほど合併頻度が高く、心不全増悪、塞栓症の誘因となる。塞栓症予防のための抗凝固療法を行いながら、発症早期であればアブレーション治療や抗不整脈薬治療によるリズムコントロールが行われるが、慢性化している症例では薬剤による心拍コントロールを行う。心室性不整脈は左室駆出率の低下した心不全（HFrEF: heart failure with reduced ejection fraction）に合併の多い不整脈であり、心臓突然死の原因となる。心臓突然死リスクの高い症例では、抗不整脈薬治療や植込み型除細動器（ICD: implantable cardioverter defibrillator）の植込み術の適応となる^3^。

これらの不整脈の診断は心電図によってなされるが、ほとんどの不整脈は間欠的に起こること、無症状であることが多いため、通常の外来診療で診断に至ることは容易ではない。不整脈の診断精度は、心電図を評価している期間（モニタリング期間）に比例することが知られており、ゆえに、最も診断精度が高いのは、患者の左前胸部の皮下に植込むことで不整脈の連続モニタリングを可能にするデバイスである、植込み型ループレコーダー（ILR: implantable loop recorder）による持続心拍モニタリングである^4^。本研究で用いる「メドトロニック LINQⅡ」は45✕8✕4.2mm、4gと小さく軽量で、モニタリングデータは遠隔モニタリングシステムを介して、医療従事者が随時、患者の心電図を確認することが可能である。本邦で既承認の医療機器であるが、本邦での適応は、1）原因不明の失神患者、2）原因不明の脳梗塞患者に限られている。一方、欧米では本邦より適応が広く、AFのモニタリング目的の使用も適応となっている。Sannaらの行った大規模試験において、通常の検査でAFが見つからなかった潜因性脳梗塞441人を無作為にILR植込み群と通常のモニタリング群の2群に割り付けて6ヶ月間観察した結果、AF検出率は8.9% vs. 1.4%と、有意にILR植込み群で高かった ^5^。これらの結果から、ILR植込みは無症候性AFのモニタリングに有用とみなされ、この目的で広く使用されている。

AFなどの不整脈のリスクが高いとされるHFrEF患者に対しても、ILRで持続心拍モニタリングを行えば、より早期の不整脈の発見、治療介入に結びつくこと、ひいてはHFrEF患者の生命予後を改善することが期待される。

本研究の対象であるHFrEF患者に対してILRによる持続心拍モニタリングを行ったデータはないため、当該患者集団において不整脈が見つかる頻度は不明である。本研究は探索的研究であり、2年間で植込み可能と思われる35症例を対象とし、ILR植込みの探索的有用性を検討する。研究対象患者はILR植込みにより不整脈の早期発見と治療介入が期待されるため参加するメリットは大きい。デメリットとしては、植込みに伴う皮膚関連合併症（感染、びらん、出血等）の可能性が考えられる。原因不明の失神患者、原因不明の脳梗塞患者、AFモニタリング目的植込み患者を対象とした海外の初期の報告ではその頻度は1.5%であり、その約半数は保存的に、残り半数はデバイス抜去で軽快している^6^。追跡期間は植込後1年として、電池寿命を勘案して植込み後3年まで観察を行う。本研究により、HFrEF症例に対するILRによる持続心拍モニタリングの有用性について有望な結果が得られた場合には、次相の開発として、製造販売企業とも協議の上、心不全患者に対する持続心拍モニタリングとしての適応拡大を目的とした臨床試験の実施等を検討する。

＜参考文献＞

1. Okura Y, et al. Impending epidemic: future projection of heart failure in Japan to the year 2055. Circ J 2008; 72: 489-491.
2. 日本循環器学会/日本心不全学会合同ガイドライン 急性･慢性心不全診療ガイドライン（2017年改訂版）
3. 日本循環器学会/日本不整脈心電学会合同ガイドライン 不整脈非薬物治療ガイドライン（2018年改訂版）
4. Aguilar M, et al. Influence of Monitoring Strategy on Assessment of Ablation Success and Postablation Atrial Fibrillation Burden Assessment: Implications for Practice and Clinical Trial Design. Circulation. 2022;145:21-30.
5. Sanna T, et al. Cryptogenic stroke and underlying atrial fibrillation. N Engl J Med. 2014;370:2478-86.
6. Mittal S, et al. Safety Profile of a Miniaturized Insertable Cardiac Monitor: Results from Two Prospective Trials. Pacing Clin Electrophysiol. 2015;38:1464-9.

# **４．臨床研究の目的**

AFと診断されていない、HFrEF患者に合併する無症候性不整脈の検出における、ILRを用いた持続心拍モニタリングの臨床的有用性を検討する。

# **５．臨床研究の内容に関する事項**

**5.1　主要評価項目および副次評価項目**

詳細は「8. 有効性の評価に関する事項」参照。

主たる解析は植込み後1年時のデータを用いて実施する。すべての症例が植込み後3年時の評価を完了したのちに最終解析を実施する。

1. **主要評価項目**

AFおよび非持続性または持続性の心室頻拍を認めた患者の割合

解析対象集団において、以下が認められた患者の割合と定義する。

- AF：6分間以上持続するもの
- 非持続性心室頻拍：心拍数150回/分を超える頻拍で、16連発以上かつ持続時間30秒以下のもの
- 持続性心室頻拍：心拍数150回/分を超える頻拍で、30秒以上持続するもの

【設定根拠】

臨床的に治療介入（薬物治療、アブレーション治療、デバイス治療）を検討する不整脈であるため

1. **副次評価項目**
   - 1. 4.5秒以上のポーズを認めた患者の割合
     2. 心拍数30回/分以下の徐脈を認めた患者の割合
     3. 高度房室ブロックを認めた患者の割合
     4. 恒久的ペースメーカー、植込み型除細動器、カテーテルアブレーション、抗不整脈薬による治療介入が行われた患者の割合
     5. 全死亡割合
     6. 心血管死亡割合
     7. 心血管疾患または心不全による入院を要した患者の割合
     8. 脳卒中を発症した患者の割合

【設定根拠】

①～③：治療介入の考慮が必要になる可能性があるため

④：ILRを用いた持続心拍モニタリングが治療に与える影響について評価するため

⑤～⑧：ILRを用いた持続心拍モニタリングの結果と臨床イベントの関連を評価するため

３）**安全性評価項目**

1. ILR植込みと関連のある有害事象の発生割合
2. 不具合の発生割合

【設定根拠】

安全性を評価する一般的な項目として設定した。

5.2　臨床研究のデザインおよびアウトライン

**5.2.1　臨床研究のデザイン**

１）研究の性質：探索的研究

２）無作為化：単一群

３）盲検化：非盲検

４）対照：非対照

５）割付：なし　

**5.2.2　臨床研究のアウトライン**

植込み後6ヶ月ごとに3年間、遠隔モニタリングシステム（CareLink）により、ILRの不整脈イベントをモニタリングする。

また、植込み前と同様に1～3ヶ月ごとの外来通院を行う。


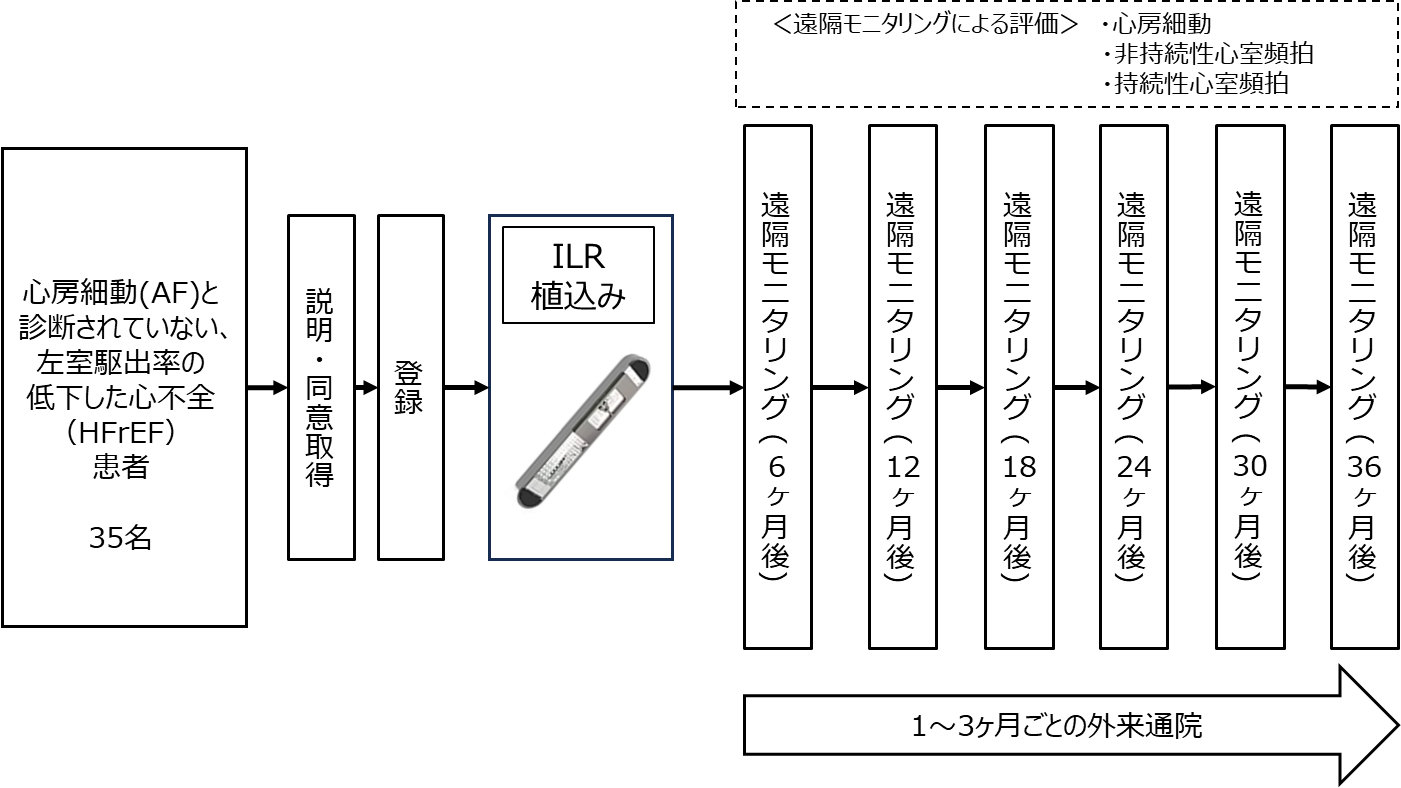


**5.3　症例登録・割付方法**

１）症例登録方法

研究責任（分担）医師は、研究対象者として文書同意を取得した患者が全ての選択基準を満たし、全ての除外基準に抵触しないことを確認する。その後、本臨床研究に従事する者は、EDCシステムに患者情報を入力し、研究対象者の登録および割付を行う。

２）割付方法

本研究では割付を実施しない。

**5.4　研究機器の概要**

本研究で使用する研究機器は以下の通りである。

「メドトロニック　Reveal LINQ モバイルマネージャ」および「メドトロニック　24967 ペイシェントコネクタ」については、既に研究実施医療機関に配備されているものを使用する。

| 一般的名称： | 植込み型心電用データレコーダ |
| --- | --- |
| 販売名： | メドトロニック　LINQⅡ |
| 分類： | 高度管理医療機器（クラスⅣ） |
| 使用目的又は効果 | 本品は、原因が特定できない、失神又は動悸等の不整脈の症状を有する患者への使用を適応とする。また、心房細動を検出するための、潜因性脳梗塞患者への使用を適応とする。  医師が必要と認めた検査で診断できない患者の皮下に植え込み、皮下心電図を記録、保存することによって不整脈の診断が可能な植込み型診断用医療機器である。 |
| 承認･認証･届出番号： | 30300BZX00278000 |
| 承認年月： | 2021年10月 |
| 製造販売業者： | 日本メドトロニック株式会社 |

※構成品として、「MyCareLink Relay Home Communicator」を含む。

| 一般的名称： | 植込み能動型機器管理用プログラム |
| --- | --- |
| 販売名： | メドトロニック　Reveal LINQ モバイルマネージャ |
| 分類： | 高度管理医療機器（クラスⅢ） |
| 使用目的又は効果： | 本品は、併用可能なメドトロニック社製植込み型心電用データレコーダ（以下、｢ICM｣という。）の1つ以上の動作モード、パラメータ等のインテロゲーション及びプログラミングを行うために用いる。  また、本品は、ICMからインテロゲートした心電図等の情報を携帯電話回線又はWi-Fiを介してWebアプリケーションサーバ（CareLink Network）へと伝送することができる。 |
| 承認･認証･届出番号： | 22800BZX00305000 |
| 承認年月： | 2016年8月 |
| 製造販売業者： | 日本メドトロニック株式会社 |

| 一般的名称： | 植込み能動型機器用プログラマ |
| --- | --- |
| 販売名： | メドトロニック　24967 ペイシェントコネクタ |
| 分類： | 高度管理医療機器（クラスⅢ） |
| 使用目的又は効果： | 本品は、併用可能なメドトロニック社製のモバイルアプリケーションと併用して、併用可能なメドトロニック社製の植込み能動型機器と非侵襲的に交信し、植込み機器をインテロゲート及び/又はプログラムするために用いる。 |
| 承認･認証･届出番号： | 30100BZX00034000 |
| 承認年月： | 2019年6月 |
| 製造販売業者： | 日本メドトロニック株式会社 |

「メドトロニック　LINQⅡ」の本邦での適応は、1）原因不明の失神患者、2）原因不明の脳梗塞患者に限られており、AFのモニタリング目的の使用は未承認である。欧米では本邦より適応が広く、AFのモニタリング目的の使用も承認されている。そのため多くの臨床研究に、AFモニタリング目的でILRが使用されている。一例として、Sannaらの行った大規模試験において、通常の検査でAFが見つからなかった潜因性脳梗塞441人を無作為にILR植込み群と通常のモニタリング群の2群に割り付けて6ヶ月間観察した結果、AF検出率は8.9% vs. 1.4%と、有意にILR植込み群で高かった^5^。

　＜使用方法の概要＞

メドトロニック　LINQⅡは45✕8✕4.2mm, 4gと小さく軽量の心電図モニタリング用の医療機器である。左前胸部の皮下に専用キットを用いて植込みを行い、専用の交信機器（ペイシェントコネクタ）とプログラマ（モバイルマネージャ）を用いて、遠隔モニタリングの設定を行う。

記録されたモニタリングデータは遠隔モニタリングシステムを介して（定期的にモニタリングデータがサーバに送信される）、医療従事者が随時、患者の心電図を確認することが可能である。

構造、原理、使用方法（植込み方法を含む）等の詳細は添付文書等を参照のこと。

**5.5　研究機器の管理の手順**

研究医療機器は、本研究に研究機器を提供する日本メドトロニック株式会社（「19．利益相反に関する事項」参照）より、研究責任医師の元に提供され、東京医科歯科大学病院の所定の場所で保管する。詳細は、別途作成する研究機器管理手順書に定める。

保管条件、使用期限等は、添付文書の定める内容に従う。

研究機器管理者：研究機器の保管、管理の責任を有するものをいう。本研究では、研究責任医師が担当する。

**5.6　研究対象者の参加予定期間**

参加予定期間：同意取得後　約３年

参加予定期間終了後、研究の実施に起因すると疑われる有害事象等が認められた場合、研究対象者の安全が確保されたと研究責任（分担）医師が判断するまでフォローアップを行う。なお、当該フォローアップ期間に関しては、研究期間には含まないものとする。

**5.7　臨床研究全体の中止基準**

研究責任医師は次の事例があった場合、研究実施継続の可否を検討する。

1. 研究機器の品質、臨床性能および安全性に関する事項、研究の実施または継続に影響を及ぼすような重要な情報、その他研究を適正に行うために重要な情報を知った場合。
2. 研究対象者の組入れが困難で、予定症例数を達成することが困難であると判断されたとき。
3. 予定症例数または予定期間終了に達する前に、研究の目的が達成されたとき。
4. 認定臨床研究審査委員会の意見として研究計画書等に対する修正の指示があり、これを受け入れることが困難な場合。
5. 認定臨床研究審査委員会が中止の判断をした場合。
6. 臨床研究法、施行規則または本研究計画書に重大なまたは継続的な違反が生じた場合。

**６．研究対象者の選択および除外関する基準**

**6.1　研究対象者（対象疾患）**

AFと診断されていないHFrEF患者

**6.2　選択基準**

以下の基準のすべてに該当する患者を、本臨床研究に組み入れる。

1. HFrEF（左室駆出率40%以下）の患者
2. HFrEFに対して適切な薬物療法が行われ、外来通院中または外来通院が可能な患者
3. CHADS_2_スコアが1点以上の患者
4. 同意取得時の年齢が20歳以上の患者
5. 本臨床研究の参加に関して患者本人から文書で同意の得られた患者

【設定根拠】

1. 本研究の対象患者群であるため設定した。
2. 適切な薬物療法により心機能が回復する可能性があるため、適切な薬物療法を行ってもHFrEFである患者を対象とした。外来通院による観察を想定しているため設定した。
3. AF検出により恩恵の大きい患者群として、AFにより血栓塞栓症を起こすリスクのある患者を設定した。
4. 個人の同意が成立する年齢および能力を考慮して、20歳以上と設定した。
5. 倫理的配慮の観点から設定した。


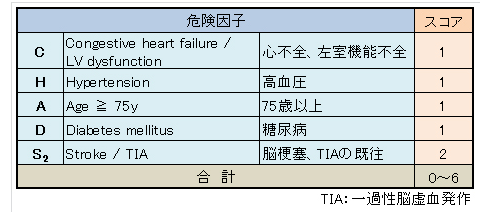


**CHADS_2_スコア**

（Gage BF, Waterman AD, Shannon W, et al. JAMA 2001; 285: 2864-2870より改変）

6.3　除外基準

以下の基準のいずれかに該当する患者は、本臨床研究に組み入れない。

1. 上室性不整脈を検出可能な植込み型心臓デバイス（CIED: cardiovascular implantable electronic device。恒久的ペースメーカー、植込み型除細動器、ILRを指す）が植込まれている患者
2. 同意取得時にAFと診断されている患者
3. 生命予後1年以内と考えられる患者
4. 免疫不全状態にある患者
5. 活動性の感染症がある患者
6. 植込み部位の皮下組織が薄く、安全にILRを植込むことが困難と判断される患者
7. その他、研究責任（分担）医師が本研究への参加が不適当であると判断した患者

【設定根拠】

1. すでに心拍連続モニタリングが可能であり、ILRを使用する意義がないため設定した。
2. 新規AFの検出が本研究の主要評価項目であるため、すでにAFと診断されている患者はILRを使用する意義がないため設定した。
3. 主たる評価時点は1年後であるため設定した。
4. ILR植込み部位の感染リスクが高いため、設定した。
5. ILR植込み部位の感染リスクが高いため、設定した。
6. 植込み自体が困難であること、また感染リスクが高いため、設定した。
7. 研究責任（分担）医師が、他の全般的要因も勘案して判断するために設定した。

# **７．研究対象者に対する治療に関する事項**


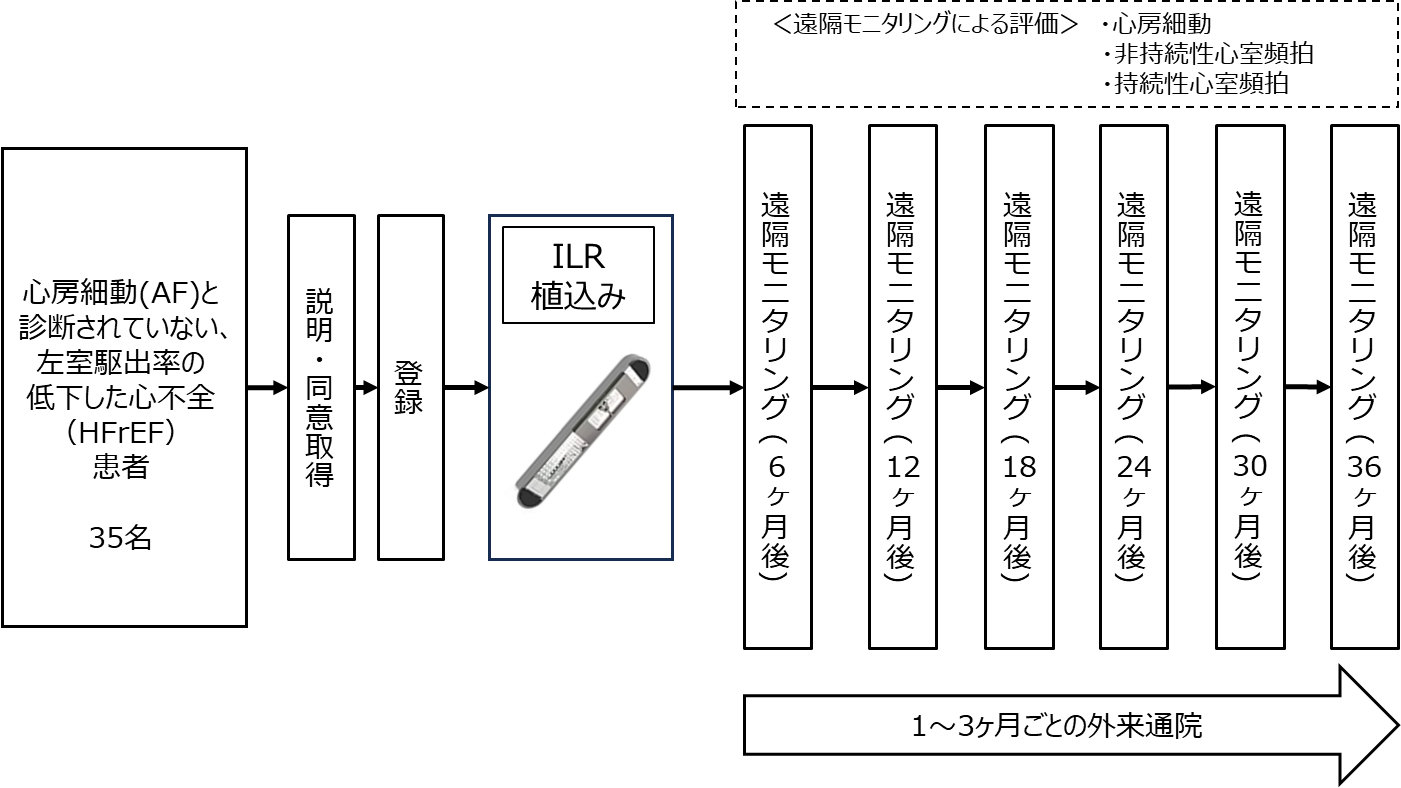
**7.1　研究治療の実施手順**

**7.1.1　同意取得～登録、登録時評価**

- 研究責任（分担）医師は、対象患者の診断およびその他の適格基準を確認した上で、文書にて本研究に関する説明を行い、本研究に対する理解を得たうえで、本人から文書同意を取得する。
- 研究責任（分担）医師は、文書同意を取得した後、登録に先立ち、患者背景情報の収集、ベースライン評価としての所定の臨床検査を行う（詳細は「7.2　観察・検査項目およびスケジュール」参照）。
- 研究責任（分担）医師は、実施すべき観察・評価を行い、研究対象者の適格性を再度確認した上で、当該研究対象者を本研究に登録する（「5.3　症例登録・割付方法」参照）。
- 適格性判定のための検査については、登録前30日以内に実施している場合は、同意取得前のデータであっても当該データを使用できるものとする。
- 登録時評価は、通常診療として行った臨床検査等の結果のうち、登録前30日以内のデータであれば、同意取得前であっても使用可とする。
- ベースラインの心電図として、ILR植込み時にモニター心電図で調律を確認する。

**7.1.2　ILRの植込み**

- 登録後30日以内に、原則として外来（外来処置室）にて循環器内科医師がILR植込みを行う。研究責任（分担）医師が入院が必要と判断した場合は、入院での植込みも許容する。30日以内にILR植込みができない場合は、プロトコール治療中止とする。
- 植込み方法の概要は以下の通り。詳細は添付文書に従う。

1. 植込み部位は第4肋間腔上（図1の部位）とする。
2. 同部位をポピドンヨードで消毒ののち、局所麻酔を行い、皮膚を挟んで持ち上げて、同梱の切開ツールの刃を押し込み切開を入れる（図2）。
3. 次に、皮下約8mmにポケットを作成できるように同梱のハンドルを押し込み（図3）、180度回転させることで切開部を開き、適切なサイズのポケットを作成する（図4）。
4. 同梱のプランジャーをハンドルに挿入し、完全に押し込む（図5）。あらかじめ装填されていた機器本体が、皮下8mm、ポケットの切開部から10ｍｍ入ったところに送達される。
5. 手で押さえながら挿入ツールを抜去する（図6）。
6. 切開創を閉じる前に、ILRから良好な心電図波形を取得できる（センシングに問題がない）ことを、メドトロニック　ペイシェントコネクタを介してプログラマ（メドトロニック　モバイルマネージャ）で確認する。問題がなければ、吸収糸で閉創する。予防的抗菌薬は投与しない。
7. プログラマで、持続心拍モニタリング（遠隔モニタリング）の各種設定（図7）を行う。

**7.1.3　遠隔モニタリング**

- 遠隔モニタリングシステムCareLink（日本メドトロニック株式会社）は、自宅に設置する附属の通信機「MyCareLink Relay Home Communicator」を介して、モニタリングデータが専用サーバに送信されるシステムである（図8）。医療機関からサーバにアクセスすることにより、不整脈イベントを確認することができる (図9にCareLinkの画面の一例を示す)。
- 外来担当医師とは別の評価担当医師が、不整脈イベント（主要評価項目、副次評価項目の①～③）について、遠隔モニタリングシステムを用いて6ヶ月ごとに、植込み後3年まで追跡調査する。各調査時点における調査結果を症例報告書に入力する。
- また、ILRの記録上で上記の不整脈イベントが検出されサーバに送信されると、評価担当医師にメールで連絡（アラート）が届く。評価担当医師は、記録された心電図をサーバ上で確認して、診断する（不整脈イベントの発生の有無を判定する）。
- 評価担当医師が、医学的に早急に外来担当医師に伝えるべき内容と判断した場合は、随時、外来担当医師に遠隔モニタリングの所見を報告する。なお、患者に不整脈を疑う症状があった場合は、随時ILRのデータを確認する。

**7.1.4　外来フォローアップ**

- ILR植込み前と同様に、1～3ヶ月ごとの外来通院を行う。その中で、副次評価項目④～⑧に該当するイベントの有無等の研究対象者の状態を含む臨床経過を確認する。
- 通常診療の中で、担当医師が必要と判断した検査等は適宜行う。
- 本研究のために追加で実施する診察、検査等はない。
- 転居等により研究実施機関への外来通院が困難となった場合には、以下の条件をすべて満たす場合に限り、本研究への参加を継続可能とする。
- 転居先で定期的に外来通院を行い、通院する医療機関から「外来フォローアップ」症例報告書の内容を診療情報提供書等の文書にて入手可能である。
- ILR抜去の際には研究実施機関に来院し、抜去を行うことに同意している。（ただし、重篤な疾患の発症等により来院不可能となった場合等については、研究責任（分担）医師と相談の上、他院での抜去または経過観察も許容する）

**7.1.5　研究の終了**

- ILR植込み後3年の時点で、本研究の観察期間を終了する。3年間の観察期間を終了した時点で、ILRを抜去する。
- 有害事象または不具合（早期の電池消耗を含む）によりILR抜去が必要となった場合は、ILRを抜去し、本研究としての観察は終了する。
- 他の植込み型心臓デバイスの植込みによりILR使用の意義がなくなった場合は、ILRを抜去し、本研究としての観察は終了する。
- 転居等により本研究としての経過観察の継続が不可能となった場合は、ILRを抜去し、本研究としての観察は終了する。
- 抜去は、植込み時と同様に原則として外来（外来処置室）にて、局所麻酔下で行う。抜去したILRは、研究実施機関の定める手順で適切に廃棄する。
- 「MyCareLink Relay Home Communicator」は、不燃ごみとして廃棄する。

**7.2　観察・検査項目およびスケジュール**

**7.2.1　スケジュール**

本研究における調査、観察および評価項目ならびに来院スケジュールは以下の通りとする。

| 観察検査項目 | 植込み前 | 植込み時 | 植込み後の遠隔モニタリング | | | | | |
| --- | --- | --- | --- | --- | --- | --- | --- | --- |
|  | 登録時検査 |  | 6ヶ月 | 12ヶ月 | 18ヶ月 | 24ヶ月 | 30ヶ月 | 36ヶ月 |
| 許容期間 |  |  | ±4週 | ±4週 | ±4週 | ±4週 | ±4週 | ±4週 |
| 同意取得 | ●※1 |  |  |  |  |  |  |  |
| 登録 | ● |  |  |  |  |  |  |  |
| 研究対象者背景 | ● |  |  |  |  |  |  |  |
| ILR植込み |  | ● |  |  |  |  |  |  |
| 遠隔モニタリング |  |  |  |  |  |  |  |  |
| 不整脈イベントの確認※2 |  |  | ● | ● | ● | ● | ● | ● |
| 心電図 | ● | ●※3 |  |  |  |  |  |  |
| 心エコー | ● |  |  |  |  |  |  |  |
| 血液検査（BNP） | ● |  |  |  |  |  |  |  |
| 胸部単純X線 | ● |  |  |  |  |  |  |  |
|  |  |  |  |  |  |  |  |  |
| 臨床経過 |  |  | 1～3ヶ月ごとの外来通院で確認 | | | | | |
| 有害事象・不具合 |  |  |  | | | | | |

● ：必須

※1：本臨床研究に関する全ての調査・観察は、研究対象者から文書による同意取得及び症例登録後に実施する。同意取得前に通常診療として実施した調査・観察・検査等の結果は、登録前30日以内であれば研究データとして利用可能とする。

※2：規定日の許容範囲は、基準来院日の前後4週間とする。

※3：ベースラインの心電図として、ILR植込み時にモニター心電図で調律を確認する。

**7.2.2　観察・検査項目**

本研究における観察・検査項目は以下の通りである。実施時期については、「7.2.1 スケジュール」に従う。

| 実施項目 | | 評価・観察項目 |
| --- | --- | --- |
| 同意取得 | | 文書による同意取得日 |
| 研究対象者背景 | 基本情報 | 生年月日、年齢、性別、身長、体重、BMI |
|  | 基礎疾患 | 心不全（なし／あり）  高血圧（なし／あり）  糖尿病（なし／あり）  脳梗塞・TIA（なし／あり）　　→　CHADS_2_スコア  血管疾患（なし／あり）　　　　→　CHA_2_DS_2_VAScスコア |
|  | 透析 | 透析（なし／あり） |
|  | 心疾患に対する手術歴 | 心臓外科手術（手術名、治療日）  心臓インターベンション（手術名、治療日） |
|  | 心不全による入院歴 | 入院歴（なし／あり）、入院回数、直近の入院日 |
|  | 使用中の  抗血小板薬・抗凝固薬  心不全治療薬  抗不整脈薬 | 抗血小板薬（なし／あり）  抗凝固薬（なし／あり）  利尿剤（なし／あり）  β遮断薬（なし／あり）  レニン・アンギオテンシン・アルドステロン阻害薬（なし／あり）  ミネラルコルチコイド受容体拮抗薬（なし／あり）  SGLT2阻害薬（なし／あり）  その他の心不全治療薬（なし／あり）  抗不整脈薬（なし／あり） |
| 植込み前（登録時検査） | 心エコー検査 | 左房径 (LAD: left atrial diameter)  左室拡張末期径 (LVDd: left ventricular end-diastolic diameter)  左室収縮末期径 (LVDs: left ventricular end-systolic diameter)  左室駆出率 (LVEF: left ventricular ejection fraction)  心室中隔厚 (IVS: interventricular septum thickness)  左室後壁厚 (LVPW: left ventricle posterior wall thickness)  その他異常所見 |
|  | 血液検査 | Brain natriuretic peptide (BNP) |
|  | 心電図検査 | 体表12誘導心電図で調律を確認する。  （洞調律／心房細動・心房粗動・心房頻拍／ペーシングリズム／その他（異所性心房調律、補充調律）） |
|  | 胸部レントゲン検査 | 心胸郭比（CTR: cardio thoracic ratio） |
| 植込み時 | 心電図検査 | モニター心電図（体表12誘導心電図も可とする）で調律を確認する。  （洞調律／心房細動・心房粗動・心房頻拍／ペーシングリズム／その他（異所性心房調律、補充調律）） |
| ILR遠隔モニタリング  （不整脈イベント） | | 評価日、評価者   - AF：6分以上持続するもの（なし／あり、ありの場合は以下） - 初回不整脈：発生日、持続時間 - 6ヶ月間での最長の不整脈：発生日、持続時間 - 6ヶ月間での不整脈の回数 - 6ヶ月間での累積持続時間（AF burden） - 非持続性心室頻拍：16連発以上で30秒以下のもの (>150bpm)（なし／あり、ありの場合は以下） - 初回不整脈：発生日、持続時間 - 6ヶ月間での最長の不整脈：発生日、持続時間 - 6ヶ月間での不整脈の回数 - 持続性心室頻拍：30秒以上持続するのもの (>150bpm)（なし／あり、ありの場合は以下） - 初回不整脈：発生日、持続時間 - 6ヶ月間での最長の不整脈：発生日、持続時間 - 6ヶ月間での不整脈の回数 - 4.5s以上のポーズ（なし／あり、ありの場合は以下） - 初回不整脈：発生日、ポーズ時間 - 6ヶ月間での最長の不整脈：発生日、ポーズ時間 - 6ヶ月間での不整脈の回数 - 30bpm以下の徐脈（なし／あり、ありの場合は以下） - 初回不整脈：発生日、持続時間 - 6ヶ月間での最長の不整脈：発生日、持続時間 - 6ヶ月間での不整脈の回数 - 高度房室ブロック（なし／あり、ありの場合は以下） - 初回不整脈：発生日、持続時間 - 6ヶ月間での最長の不整脈：発生日、持続時間 - 6ヶ月間での不整脈の回数 |
| 臨床経過 | | - 死亡（なし／あり、死亡日、心血管死亡／その他の死亡、詳細） - 心血管疾患または心不全による入院（なし／あり、入院日、詳細） - 脳卒中（なし／あり、発生日、詳細） - 恒久的ペースメーカー植込み（なし／あり、植込み日、詳細） - 植込み型除細動器による治療（なし／あり、実施日、詳細） - カテーテルアブレーション（なし／あり、実施日、詳細） - 抗不整脈薬による不整脈治療（なし／あり、実施日、詳細） |
| 有害事象 | | 問診および身体診察により、有害事象の有無、内容等を確認する。  発生時：事象名、発現時期・消失時期、程度（Grade）、処置、転帰、重篤性（重篤／非重篤）、予測可能性（既知・予期される／未知・予期されない）、ILRとの関連性、有害事象によるILR抜去（なし／あり、実施日、詳細）等 |
| 不具合 | | 研究機器の不具合の有無、内容等を確認する。  発生時：不具合名、発現時期・消失時期、有害事象の有無、予測可能性（既知・予期される／未知・予期されない）、不具合によるILR抜去（なし／あり、実施日、詳細）等 |

**CHA2DS2-VAScスコア**

（Camm AJ, Kirchhof P, Lip GY, et al. Eur Heart J 2010; 31: 2369-2429より改変）


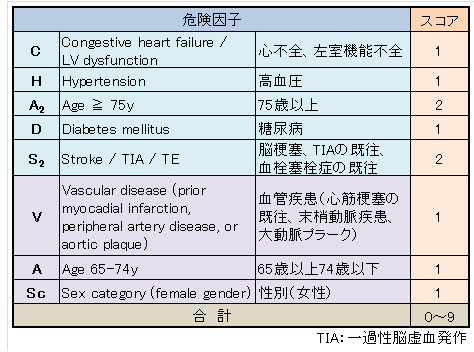


**7.3　併用薬（療法）に関する規定**

**7.3.1　併用可能・制限薬（療法）**

- MRI検査：原則として添付文書13の条件下で行うこと。

**7.3.2　併用禁止薬（療法）**

- ジアテルミー（高周波、短波、マイクロ波）治療器
- 恒久的ペースメーカー植込み
- 植込み型除細動器の植込み

**7.4　研究対象者への指導事項**

研究責任（分担）医師は、研究開始前に研究対象者に対して以下の指導を行う。

１）他科・他院を受診する際は、研究に参加していることを当該医師に告げるとともに、可能な限り事前に研究責任（分担）医師に相談するよう指導する。

なお、事前に相談ができなかった場合は、事後に必ず研究責任（分担）医師に報告するよう指導する。

２）体調不良が認められた場合は速やかに研究責任（分担）医師に報告し、受診の必要性について相談するよう指導する。

３）MRI検査を行う際は、事前に担当医師に連絡するよう指導する。

４）高周波、短波、マイクロ波を用いた治療器の使用は避けるよう指導する。

５）嗜好品や運動については研究責任（分担）医師の指示に従うよう指導する。

６）本臨床研究に関する情報を、SNS等に公開しないよう、指導する。

７）本研究に参加している期間中に研究対象者本人が妊娠した場合、ただちに研究責任（分担）医師に報告するよう指導する。

８）併用禁止薬（療法）および併用制限薬（療法）を遵守するように指導する。

**7.5　研究終了後の対応**

研究対象者が、研究参加終了後においても研究の結果により得られた最善の予防、診断および治療を受けることができるように努める。

本研究としての観察が終了した後、ILRによる持続心拍モニタリングの継続を希望する患者については、研究用に植込みを行ったILRを抜去の上、自費診療としてILR再植込み・持続心拍モニタリングを行う。

**7.6　研究対象者ごとの中止基準**

以下の中止基準に該当する場合は、当該研究対象者の研究参加を中止する。

1. 研究対象者の自由意思による同意撤回の申し入れがあった場合
2. 登録後30日以内にILR植込みができなかった場合
3. 研究責任医師または研究分担医師により、原疾患あるいは合併症の悪化または重篤な有害事象（または疾病等）の発生のため研究の継続が困難と判断された場合
4. 有害事象または不具合により、ILRを抜去した場合
5. 不整脈に対し、恒久的ペースメーカー植込み等のデバイス治療が必要となり、ILRを抜去した場合
6. 転居等により本研究としての経過観察の継続が不可能となり、ILRを抜去した場合
7. 併用禁止薬（療法）を投与・実施する必要性が生じた場合または投与・実施した場合
8. 臨床研究法および施行規則違反、選択基準違反または除外基準違反など、研究計画書からの重大な逸脱が判明した場合
9. 研究計画書の遵守が不可能になった場合

10) 研究対象者の妊娠が判明した場合

11）研究全体が中止された場合

12）その他、研究責任医師または研究分担医師により研究の継続が困難と判断された場合

【設定根拠】

研究を倫理的に実施するため、また、研究対象者の安全性に配慮して設定した。

【中止時の対応方法】

研究責任医師および研究分担医師は、中止基準に該当するため研究を中止した場合には、当該研究対象者に対し適切な措置を講じ、中止の日付・時期、理由、経過等を診療記録等に明記する。

なお、研究治療開始後に同意の撤回があった場合は、その原因が医薬品等の効果不発揮または有害事象（または疾病等）によるものか、あるいは偶発的事象（転居など）によるものかをできるだけ明らかにする。

臨床的に意義のある異常（臨床検査値を含む）が認められる場合は、適切な検査を行い、医学的に許容できる範囲に戻るまで、あるいは研究責任医師および研究分担医師が追跡調査の必要がないと判断するまで、追跡調査を行う。研究中止後も持続する有害事象（または疾病等）については、事象が消失するか、研究責任医師および研究分担医師が追跡調査の必要がないと判断するまで、追跡調査を実施する。

**８．有効性の評価に関する事項**

**8.1　有効性の評価指標**

**8.1.1　主要評価項目（Primary endpoint）**

AFおよび非持続性または持続性の心室頻拍を認めた患者の割合

**8.1.2　副次評価項目（Secondary endpoint）**

1. 4.5秒以上のポーズを認めた患者の割合
2. 心拍数30回/分以下の徐脈を認めた患者の割合
3. 高度房室ブロックを認めた患者の割合
4. 恒久的ペースメーカー、植込み型除細動器、カテーテルアブレーション、抗不整脈薬による治療介入が行われた患者の割合
5. 全死亡割合
6. 心血管死亡割合
7. 心血管疾患または心不全による入院を要した患者の割合
8. 脳卒中を発症した患者の割合

**8.2　有効性評価指標に関する評価、記録**

主たる解析は植込み後1年時のデータを用いて実施する。すべての症例が植込み後3年時の評価を完了したのちに最終解析を実施する。

１）主要評価項目

AFおよび非持続性または持続性の心室頻拍を認めた患者の割合

［定義］

解析対象集団において、ILRの遠隔モニタリングで記録された心電図上で、以下が認められた患者の割合と定義する。

- AF：6分間以上持続するもの
- 非持続性心室頻拍：心拍数150回/分を超える頻拍で、16連発以上かつ持続時間30秒以下のもの
- 持続性心室頻拍：心拍数150回/分を超える頻拍で、30秒以上持続するもの

２）副次評価項目

1. 4.5秒以上のポーズを認めた患者の割合
2. 心拍数30回/分以下の徐脈を認めた患者の割合
3. 高度房室ブロックを認めた患者の割合

［定義］

解析対象集団において、ILRの遠隔モニタリングで記録された心電図上で、各々の不整脈イベントが認められた患者の割合と定義する。

1. 恒久的ペースメーカー、植込み型除細動器、カテーテルアブレーション、抗不整脈薬による治療介入が行われた患者の割合
2. 全死亡割合
3. 心血管死亡割合
4. 心血管疾患または心不全による入院を要した患者の割合
5. 脳卒中を発症した患者の割合

［定義］

解析対象集団において、各々の臨床イベントが認められた患者の割合と定義する。

**8.3　有効性評価指標に関する解析の方法ならびに時期**

「10．統計的な解析に関する事項」参照。

**９．安全性の評価に関する事項**

**9.1　安全性の評価指標**

1. ILR植込みと関連のある有害事象の発生割合
2. 不具合の発生割合

有害事象とは、実施された研究との因果関係の有無を問わず、研究対象者に生じたすべての好ましくないまたは意図しない傷病若しくはその徴候をいう。

不具合とは、機器の破損、作動不良等広く品質、安全性、性能等に関する機器の具合がよくないことをいい、設計、交付、保管、使用のいずれの段階によるものであるかを問わない。なお、本研究における不具合とは、有害事象に該当しないものとする。不具合により発生した有害事象は、有害事象として報告する。

また、本試験では、心疾患以外の登録前から発現している症状や疾病は併存疾患として取り扱い、悪化の有無にかかわらず有害事象としない。加えて、研究参加中に認められた以下の事象は有害事象とは取り扱わず、報告対象外とする。

- 植込み後７日間以内の、感染が疑われない発熱
- Grade 3以下の臨床検査値の異常（臨床症候を伴わないもの）

※臨床症候（例：感染、出血など）による二次的な臨床検査値の異常については有害事象としての報告は

不要とし、検査値異常の要因となっている臨床症候を有害事象として報告する。

- 感冒
- Grade 2以下の食欲不振、便秘、下痢、嘔吐
- 不整脈を含む心疾患ならびにそれらに伴う症状（失神、胸痛等）と明らかに関連のない外傷

**9.2　安全性評価指標に関する評価、記録、解析の方法ならびに実施時期**

１）有害事象発生割合

研究機器（ILR）が使用されたすべての研究対象者のうち、有害事象（または疾病等）が認められた者の割合と定義する。評価の対象とする期間は、ILR植込み日から3年間とする。

有害事象（または疾病等）の事象名、発現時期・消失時期、程度（Grade）、処置、転帰、重篤性（重篤／非重篤）、研究機器との関連性、予測可能性（既知・予期される／未知・予期されない）等を、診療記録および症例報告書に記載する。

２）程度の判定基準

程度は、有害事象共通用語規準（Common Terminology Criteria for Adverse Events：CTCAE）version5.0に基づき判定する。

Grade 1：軽症、無症状または軽度の症状がある、臨床所見または検査所見のみ、治療不要

Grade 2：中等症、最小限・局所的・非侵襲的治療を要する、年齢相応の身の回り以外の日常動作の制限

Grade 3：重症または医学的に重大であるが直ちに生命を脅かすものではない、入院または入院期間の延長を要する、活動不能・動作不能、身の回りの日常生活動作の制限

Grade 4：生命を脅かす、緊急処置を要する

Grade 5：有害事象による死亡

３）重篤性の判定基準

重篤性は以下の基準で判定する。

（１）重篤

①　死亡

②　死亡につながるおそれのあるもの

③　治療のために医療機関への入院または入院期間の延長が必要とされるもの

④　障害

⑤　障害につながるおそれのあるもの

⑥ ①から⑤に準じて重篤であるもの

⑦ 後世代における先天性の疾病または異常

（２）非重篤

上記の「重篤」以外のもの

**9.3　安全性評価指標に関する解析の方法ならびに実施時期**

　「10．統計的な解析に関する事項」参照。

**9.4　疾病等の情報収集、記録および報告に関する手順**

　　　本研究における疾病等が発生した場合の対応に関する手順は、以下の通りとする。

**9.4.1　疾病等に関する定義**

１）疾病等

疾病等とは、有害事象のうち、臨床研究の実施に起因すると疑われる疾病、障害若しくは死亡または感染症に加え、臨床検査値の異常や諸症状を含むものを指す。

２）予測可能性

疾病等報告に際し、研究対象の医療機器における疾病等の発生あるいは発生数、発生頻度、発生条件等の発生傾向が、研究計画書、研究機器の概要を記載した書類、同意説明文書、添付文書の該当箇所等から予測できるものを「既知」、予測できないものを「未知」とする。

３）因果関係

GCP省令のガイダンスを参考に、少なくとも合理的な可能性について研究対象の研究機器との関連性を判定する。本研究との因果関係が否定できない場合は「因果関係あり」とする。

４）不具合

不具合とは、機器の破損、作動不良等広く品質、安全性、性能等に関する機器の具合がよくないことをいい、設計、交付、保管、使用のいずれの段階によるものであるかを問わない。なお、本研究における不具合とは、有害事象に該当しないものとする。不具合により発生した有害事象は、有害事象として報告する。

**9.4.2　疾病等報告の対象および報告期間**

研究責任医師は、本研究の実施について、下表に掲げる緊急報告が必要な疾病等または不具合の発生を知ったときは、それぞれに定める期間内にその旨を実施医療機関の管理者に報告するとともに、認定臨床研究審査委員会に報告する。また、当該疾病等が厚生労働大臣への報告を要する事象である場合は、あわせて厚生労働大臣にも定める期間内に報告する。さらに、研究対象医療機器を製造販売しようとする企業（医薬品等製造販売業者）にも情報提供を行う。

| 疾病等報告の報告対象と報告期限 | | | | | | | | |
| --- | --- | --- | --- | --- | --- | --- | --- | --- |
| 因果関係 | | | 因果関係あり | | | | 因果関係なし | |
| 報告先 | | | 厚生労働大臣 | | CRB（認定臨床研究審査委員会）  （規則第54・55条による） | | CRB  （※2） | |
|  |  |  | （PMDA） | (地方厚生局) |  |  |  |  |
| 報告の種類 | | | 期限内報告 | 定期報告 | 期限内報告 | 定期報告 | 期限内報告 | 定期報告 |
| 医薬品等 | 未知 | 死亡・死亡のおそれ | ○ | ○ | 7日 | ○ |  | ○ |
|  |  | その他重篤※1 | ○ | ○ | 15日 | ○ |  | ○ |
|  |  | 非重篤 |  | ○ |  | ○ |  | ○ |
|  | 既知 | 死亡・死亡のおそれ |  | ○ | 15日 | ○ |  | ○ |
|  |  | その他重篤※1 |  | ○ | 30日(※2) | ○ |  | ○ |
|  |  | 非重篤 |  | ○ |  | ○ |  | ○ |
| 不具合 | 未知 | 死亡のおそれ |  |  | 30日 |  |  |  |
|  |  | 重篤のおそれ |  |  | 30日 |  |  |  |
|  |  | 非重篤のおそれ |  |  |  |  |  |  |
|  | 既知 | 死亡のおそれ |  |  | 30日 |  |  |  |
|  |  | 重篤のおそれ |  |  | 30日 |  |  |  |
|  |  | 非重篤のおそれ |  |  |  |  |  |  |

※1：「その他重篤」とは、9.2 ２）重篤性の判定基準(1)の③～⑦である。

※2：本研究では、東京医科歯科大学臨床研究審査委員会が承認した疾病等・不具合報告の取扱いに関する標準業務手順書に定める運用に従う。

**9.4.3　疾病等報告に関する手順**

１）疾病等発生時の手順

（１）本研究に従事する者および研究分担医師（以下「研究分担医師等」という。）は、疾病等の発生を知り得たら、研究対象者に適切な処置を施し、最善の策を講じると共に、診療記録やCRFに記載する。

（２）当該研究責任医師は、知り得た疾病等が9.4.2の表に掲げる疾病等報告のうち緊急報告の対象となる場合には、施設の手順に従って実施医療機関の管理者へ報告する。まず、当該事象の発生を知り得てから、速やかに報告（第一報）をする。

① 認定臨床研究審査委員会への緊急報告の対象となる場合

（ⅰ）臨床研究法施行規則第54・55条に該当する場合

研究責任医師は、「医療機器の疾病等または不具合報告書　統一書式9」および「統一書式 詳細記載用書式」を作成する。なお、上記①で厚生労働大臣に報告を行う場合には、「疾病等報告書（医薬品）別紙様式2-1」を添付することでもよい。研究責任医師は、定められた報告期限内に、「臨床研究審査委員会　申請システム」を用いて認定臨床研究審査委員会に報告を行う。

（ⅱ）（ⅰ）以外（東京医科歯科大学臨床研究審査委員会が承認した疾病等・不具合報告の取扱いに関する標準業務手順書による報告対象の事項）に該当する場合

　　（ⅰ）と同様の手順で報告を行う。

② 厚生労働大臣への緊急報告の対象となる場合

研究責任医師は、「jRCT臨床研究等提出・公開システム」上の「疾病等報告書（医療機器）別紙様式2-2」に入力を行い、十分確認を行ったうえで、定められた報告期限内に「jRCT臨床研究等提出・公開システム」を用いてPMDAに送信をする。

（３）研究責任医師は、「臨床研究審査委員会　申請システム」を用いて実施医療機関の管理者に報告を行う。また、上記（２）①（ⅰ）に該当する場合には、研究責任医師は当該疾病等を製造販売業者にも情報提供を行う。

（４）発生した疾病等の追加報告を実施する場合には、上記（１）～（３）と同様の手順にて対応をする。第1報では、認定臨床研究審査委員会への報告期間内に、それまでに判明している範囲内で報告することとし、その後続報として速やかに詳細な要因等を報告する。なお、当該続報については必ずしも報告期限内でなくとも差し支えない。

２）定期報告の手順

　（１）研究責任医師は、臨床研究法施行規則第60条に従い、本研究における臨床研究法第13条に基づく疾病等の報告件数を定期報告として厚生労働大臣に報告するために、「jRCT臨床研究等提出・公開システム」を用いて「定期報告書　別紙様式3」を作成する。なお、当該疾病等の報告件数は、本研究の対象者の症例数ではなく、事象の件数を記入する。

（２）研究責任医師は、臨床研究法施行規則第59条に従い、本研究に係る疾病等の発生状況およびその後の経過を報告するために「定期報告書　統一書式5」を作成し、上記（１）の「定期報告書　別紙様式3」を添付して、実施医療機関の管理者に報告するとともに、「24. 定期報告」の項で定めた期間内に認定臨床研究審査委員会へ報告を行う。9.4.2の表に掲げる定期報告に該当する疾病等が複数ある場合は、必要に応じて一覧表を作成し、「定期報告書　統一書式5」に添付する。なお、非重篤な疾病等については、高頻度に発生している疾病等や通常の診療に比べて特筆すべき事項などを報告対象とすることで差し支えない。

（３）研究責任医師は、認定臨床研究審査委員会の承認を得て、「24. 定期報告」の項で定めた期間内に「jRCT臨床研究等提出・公開システム」を用いて地方厚生局に定期報告を届け出る。その他、定期報告に関する事項は「24. 定期報告」の項に従い対応する。

**9.5　疾病等発生後の研究対象者の観察**

有害事象（または疾病等）発生後の研究対象者の観察については、有害事象（または疾病等）が消失・回復するまで、あるいは研究責任医師または研究分担医師が追跡の必要がないと判断するまで、追跡調査を行う。

**10．統計的な解析に関する事項**

本研究の統計解析計画の概要を以下に示す。

**10.1　解析対象集団**

**10.1.1　最大の解析対象集団 (Full Analysis Set, FAS)**

本研究に登録され、ILR（研究機器）植込み術が行われたすべての研究対象者から、治療後の評価データが全くない症例を除いた集団と定義する。

**10.1.2　研究計画書に適合した対象集団 (Per Protocol Set, PPS)**

FASから、研究方法や併用療法など研究計画書の規定に対して、以下に示すような重大な違反があった症例を除いた研究対象者の集団と定義する。

- 選択基準違反
- 除外基準違反
- 併用禁止薬（療法）違反

**10.1.3　安全性解析対象集団 (Safety Analysis Set, SAS)**

本研究に登録され、ILR（研究機器）植込み術が行われたすべての研究対象者の集団と定義する。

**10.2　目標登録数と設定根拠**

目標登録数：35例

【設定根拠】

本研究の対象であるHFrEF患者に対してILRによる持続心拍モニタリングを行ったデータはないため、当該患者集団において上記の不整脈が見つかる頻度は不明である。本研究は探索的研究であり、統計学的根拠に基づく目標症例数の設定を行わないこととし、研究の実施期間を考慮して、実施可能性の観点から、2年間で植込み可能と思われる35例を目標登録数に設定した。

**10.3　症例の取扱い**

原則として登録された症例については、研究責任医師と統計解析責任者等が協議の上、症例の取扱いを決定する。新たな問題が起こった場合の症例の取扱いについても、研究責任医師および統計解析責任者等が協議の上決定し、いずれの場合も、症例取扱いの決定内容について記録に残す。

**10.4　データの取扱い**

データ集計・解析時におけるデータの取扱いについては、原則として以下に示す通りとする。疑義が生じた場合は、研究責任医師および統計解析責任者が協議の上、決定する。欠測値の補完は行わない。

**10.5　統計解析項目および解析計画**

主たる解析は、植込み後1年時のデータを用いて実施する。すべての症例が植込み後1年時の評価を完了したのちに、主たる解析を実施する。なお、すべての症例が植込み後3年時の評価を完了したのちに最終解析を実施する。

全ての有効性評価において、FASにおける解析を主たる解析とし、参考としてPPSにおける解析を行う。

安全性評価として、SASにおける解析を実施する。

**10.5.1　研究対象者の背景の要約**

各解析対象集団における研究対象者の背景データの分布および要約統計量を算出する。名義変数および順序変数については、カテゴリの頻度および割合を示す。連続変数については要約統計量（例数、平均値、標準偏差、最小値、中央値、最大値）を算出する。

**10.5.2　主要評価項目の解析**

主要評価項目：AFおよび非持続性または持続性の心室頻拍を認めた患者の割合

FASにおける解析を主たる解析とし、参考としてPPSにおける解析を行う。

該当する不整脈イベント（「8.1.1 主要評価項目」参照）を発生した研究対象者の割合およびその両側95%信頼区間を算出する。内訳として、各々の不整脈イベント（AF、非持続性心室頻拍、持続性心室頻拍）の割合およびその両側95%信頼区間も算出する。信頼区間の算出にはClopper-Pearson法を用いる。

副次解析として、Kaplan-Meier法を用いて、ILR植込み日から該当する不整脈イベント発生日、および各々の不整脈イベント（AF、非持続性心室頻拍、持続性心室頻拍）までの期間の生存関数をそれぞれ図示する。なお、イベントを起こさなかった研究対象者は最終観察日をもって打ち切りとし、追跡不能例は追跡不能となる以前でイベントを起こしていないことが確認された最終日をもって打ち切りとする。また、各遠隔モニタリング時点における該当する不整脈イベントおよび各々の不整脈イベントを起こしていない研究対象者の割合とその両側95%信頼区間をそれぞれ算出する。信頼区間の算出にはGreenwoodの公式を用いる。

**10.5.3　副次評価項目の解析**

① 4.5秒以上のポーズ

② 心拍数30回/分以下の徐脈

③ 高度房室ブロック

FASにおける解析を主たる解析とし、参考としてPPSにおける解析を行う。

該当する不整脈イベント（「8.1.2 副次評価項目」参照）を発生した研究対象者の割合およびその両側95%信頼区間を算出する。信頼区間の算出にはClopper-Pearson法を用いる。併せて、経時的な不整脈イベントの発生状況について、Kaplan-Meier法を用いて提示する。

副次解析として、Kaplan-Meier法を用いて、ILR植込み日から該当する各不整脈イベント発生日までの期間の生存関数をそれぞれ図示する。また、各遠隔モニタリング時点における該当する各不整脈イベントを起こしていない研究対象者の割合とその両側95%信頼区間をそれぞれ算出する。信頼区間の算出にはGreenwoodの公式を用いる。

④　恒久的ペースメーカー、植込み型除細動器、カテーテルアブレーション、抗不整脈薬による治療介入が行われた患者の割合

⑤ 全死亡割合

⑥ 心血管死亡割合

1. 心血管疾患または心不全による入院を要した患者の割合
2. 脳卒中を発症した患者の割合

FASにおける解析を主たる解析とし、参考としてPPSにおける解析を行う。

該当する臨床イベント（「8.1.2 副次評価項目」参照）を発生した研究対象者の割合およびその両側95%信頼区間を算出する。信頼区間の算出にはClopper-Pearson法を用いる。併せて、⑤・⑥・⑧については、経時的な臨床イベントの発生状況について、Kaplan-Meier法を用いて提示する。

副次解析として、⑤・⑧については、Kaplan-Meier法を用いて、ILR植込み日から該当する各臨床イベント発生日までの期間の生存関数をそれぞれ図示し、各遠隔モニタリング時点における該当する各臨床イベントを起こしていない研究対象者の割合とその両側95%信頼区間をそれぞれ算出する。信頼区間の算出にはGreenwoodの公式を用いる。⑥については、心血管死亡以外の死亡を競合リスクとして、ILR植込み日から該当する心血管死亡日までの期間の累積発生関数を図示し、各遠隔モニタリング時点における該当する臨床イベント発生割合とその両側95%信頼区間を算出する。信頼区間はCounting process法によって推定される標準誤差に基づいて算出する。

**10.5.4　安全性評価項目の解析**

1. ILR植込みと関連のある有害事象の発生割合
2. 不具合の発生割合

安全性評価の対象集団はSASとし、

1. 研究機器植込み後に新たに発現した、ILR植込みと関連ありと判定された有害事象を、重症度別に発現頻度と発現割合を集計する。
2. 研究機器の不具合を、内容別に発現頻度と発現割合を集計する。

**10.5.5　中間解析**

本研究では中間解析を実施しない。

**10.6　最終解析**

最終植込み症例のILR植込み日から3年後を以て観察期間終了とする。3年間の観察期間終了後、データが得られた症例が固定された後に、最終解析を行う。統計解析責任者が「解析報告書」をまとめ、研究責任医師に提出する。

**11．原資料等の閲覧に関する事項**

本研究における原資料とは、診療録、各種検査データ、投薬記録等とする。

研究責任医師および実施医療機関の管理者は、本研究に関するモニタリングおよび監査ならびに認定臨床研究審査委員会および規制当局による調査を受け入れ、その際に、本研究に関する原資料等の全ての資料を直接閲覧に供することを保証する。

診療記録に記載が無く症例報告書に記載されたデータのうち、以下に示す記載項目は、症例報告書の記載を原資料とする。

１）併用薬（または療法）の使用目的、併用療法の実施目的

２）有害事象（または疾病等）の重篤度、程度、転帰、転帰日、研究機器との因果関係、研究機器との因果関係を判定した理由

３）中止日、中止理由、中止の原因となった有害事象または不具合、中止後の経過および追跡調査の結果

４）研究責任（分担）医師コメント

**12．品質管理および品質保証に関する事項**

**12.1　モニタリング**

研究責任医師は、研究の品質管理を目的に、本研究のモニタリングに関する手順書を作成し、研究計画書とともに認定臨床研究審査委員会の審議、承認を受ける。また、モニタリングを担当するモニタリング担当者を指名する。モニタリング担当者はモニタリング手順書に従い、研究期間を通じて本研究が最新の研究計画書および規制要件（臨床研究法、施行規則等）を遵守して実施されていることを確認し、その確認した結果をモニタリング手順書に従い研究責任医師に報告する。また、モニタリング担当者は、モニタリングの際に得た研究対象者の個人情報を漏らしてはならない。

**12.2　監査**

本研究の監査は、東京医科歯科大学 ヘルスサイエンスR&Dセンターが実施する。

研究代表医師は、臨床研究法および施行規則等の適用されるすべての規制要件、研究計画書および研究の実施に係る手順書を遵守して、研究の実施ならびにデータの作成、記録および報告が行われていることを保証するために、独立した立場の者に監査を行わせる。

監査の具体的な方法等については、別途定める監査手順書に従うものとする。

# **13．倫理的な配慮に関する事項**

**13.1　法令等の遵守**

本研究に関係するすべての研究者は「ヘルシンキ宣言」（日本医師会訳）^1）^および「臨床研究法」（平成29年法律第16号）^2）^「臨床研究法施行規則」（平成30年厚生労働省令第17号）ならびに関連通知に従って本試験を実施する。

1） <http://dl.med.or.jp/dl-med/wma/helsinki2013j.pdf>

2） <http://www.mhlw.go.jp/stf/seisakunitsuite/bunya/0000163417.html>

**13.2　予期される利益、負担および不利益**

１）予期される利益

本研究に参加することにより研究対象者に直接の利益は生じない。研究成果により将来の医療の進歩に貢献できる可能性がある。

２）予期される不利益

研究対象医療機器「メドトロニック　LINQ II」は、高度管理医療機器（クラスⅣ）として、本邦において承認されている医療機器である。本研究の研究対象者に対しては適応外使用となるが、使用方法は添付文書のとおりであること、心電図の測定機器であること、植込む部位は皮下であること、等を踏まえると、研究対象者の負担は限定的であり、研究に伴い重大な身体的リスクが生じる可能性は低いと考えている。

添付文書では電池早期消耗、センシング不全、データ収集機能不全などの不具合（頻度不明）、機器の体外への露出、血腫、出血等の有害事象（頻度不明）が報告されている。その他、未知および既知の副作用または不具合が発生する可能性が否定できない。副作用または不具合の発生が認められた場合は、研究に従事する者は適切な処置を行うと共に、最善の治療を尽くす。研究責任医師は、安全性に関する情報を収集し、必要に応じて研究計画書を改訂する等の適切な対応を行う。

３）予期される負担

本研究に参加することによる、来院回数、検査回数、診察・検査時間などは、通常診療と同程度である。なお、本研究において研究機器を使用することによる研究対象者の費用負担は発生しない。（「15.　金銭の支払いおよび補償に関する事項」参照）。

４）リスクを最小化する方策

本研究では、外来通院可能な患者を研究対象者としているため、研究に伴う身体的リスクが生じる可能性は低いと考えているが、各来院時において、研究責任（分担）医師は、研究対象者の状態に十分に注意し、有害事象・不具合の早期発見に努める。

**13.3　研究対象者に係わる遺伝的特徴等に関する研究結果や偶発的初見の取扱い**

本研究では研究対象者の健康、遺伝学的特徴に関する重要な知見が得られるような検査・解析は実施しない。

# **14．記録（データを含む）・試料の取扱いおよび保存に関する事項**

**14.1　データ収集の方法**

本研究では、研究データについては、電子データ管理システム（EDCシステム）「eACReSS」に入力し、管理する。入力された情報は、東京医科歯科大学のeACReSSサーバ内に保管される。

＊「eACReSS」は、大学病院臨床試験アライアンス事業にて整備された臨床研究データを管理する専用システムである。大学病院臨床試験アライアンスは、関東甲信越地区の8つの国立大学からなる治験・臨床研究のアライアンスである。(東京大学、千葉大学、東京医科歯科大学、筑波大学、群馬大学、新潟大学、信州大学、山梨大学)

① 保管場所：東京医科歯科大学「eACReSS」サーバ内

② 保管責任者：村上 妙子（東京医科歯科大学 ヘルスサイエンスR&Dセンター）

③ 保存期間：研究終了後10年間

④ 廃棄方法：データは復元不可能な状態に処理して廃棄する。

⑤ 二次利用の可能性：　□なし　　■あり（「21.2 データの二次利用」参照）

**14.2　記録の保存**

１）本研究に関する保存すべき記録は、次に掲げる事項とする。

（１）研究対象者ごとに医薬品等を用いた日時および場所

（２）研究対象者を特定する事項

（３）研究対象者に対する診療および検査に関する事項

（４）本研究への参加に関する事項

（５）前各号のほか、本研究を実施するために必要な事項

２）研究責任医師は、本研究が終了した日から10年間、本研究に関する保存すべき記録を次に掲げる書類とともに保存する。

（１）研究計画書、実施計画、研究対象者に対する説明およびその同意に係る文書、総括報告書その他の臨床研究法、施行規則の規定により研究責任医師が作成した文書またはその写し

（２）認定臨床研究審査委員会から受け取った審査意見業務に係る文書

（３）モニタリングおよび監査（監査を実施する場合）に関する文書

（４）原資料等

（５）本研究の実施に係る契約書（医薬品等製造販売業者またはその特殊関係者との契約の締結の規定（臨床研究法第三十二条）により締結した契約に係るものを除く）

（６）本研究に用いる医薬品等の概要を記載した文書、および本研究に用いる医薬品等の製造・入手・処分に関する文書等

（７）前各号のほか、本研究を実施するために必要な文書

３）研究責任医師は、本研究に関する保存すべき記録の事項（１）～（５）の修正を行う場合は、修正者の氏名および修正を行った年月日を記録し、修正した記録とともに保存する。

**14.3　試料の保管等**

本研究では、研究対象者から試料（血液、組織、細胞、体液、排泄物及びこれらから抽出したDNA等）を採取・使用・保管する予定はない。

**14.4　情報の保管と廃棄**

「eACReSS」で管理する以外の研究等の実施に係わる文書の保存は、循環器内科学分野の研究室の研究者以外がアクセス不可能な、施錠可能な保管庫で行うものとし、研究終了後10年間保管する（保管責任者：研究責任医師）。その他事項は各実施医療機関の手順に従い適切に保存する。

また、本研究で収集する情報やデータは、氏名、イニシャル、患者IDなどの個人情報をはずし、新たな符号をつけて個人が識別できないようにした上で、研究責任医師の責任のもとで適切に保管する。

保存期間終了後に廃棄する場合は、電子データは復元不可能な状態に処理して廃棄し、紙資料については個人情報に注意しシュレッダーで裁断する他、別途各実施医療機関に手順がある場合はその手順に従い適切に廃棄する。

**15．金銭の支払いおよび補償に関する事項**

**15.1　金銭の支払い（研究対象者の費用負担）**

本研究では、製造販売企業（日本メドトロニック株式会社）から提供を受けた研究医療機器を使用するため、本研究において研究医療機器を使用（ILR植込みおよび遠隔モニタリング）することによる研究対象者の費用負担は発生しない。本研究で実施する検査・診察に伴う研究対象者の費用負担は、通常診療で発生する費用と同様である。

**15.2　補償に関する事項**

研究責任医師、研究分担医師および実施医療機関は、本研究の実施に起因して研究対象者に健康被害が発生した場合には、研究対象者がただちに適切な診断、治療および必要な措置を受けることができるよう医療の提供その他の必要な措置を講ずる。

研究責任医師は、当該健康被害によって研究対象者が被った損失を補うために、以下の補償内容を有する臨床研究保険に加入し、臨床研究保険の支払条件に従って補償を行う。ただし、研究対象者に過失がある場合は対象とはならない。

・ 研究対象者の死亡または後遺障害に対する補償金

・ 研究対象者の健康被害の治療のために要する医療費・医療手当

また、研究責任医師および研究分担医師は、本研究における通常範囲の医療行為に起因する研究対象者の健康被害に備えて医師賠償責任保険にも必ず加入する。

**16．情報の公表**

本研究を実施する場合には、あらかじめ、本研究を実施するに当たり世界保健機関が公表を求める事項その他の臨床研究の過程の透明性の確保および国民の臨床研究への参加の選択に資する事項をjRCTに記録し、当該事項を公表するものとする。jRCT公表後に研究を開始するが、実施計画の変更、研究の進捗に応じて適宜情報を更新し、また、主要評価項目報告書または総括報告書を作成した場合は、主要評価項目報告書または総括報告書の概要も公表する。

**17．実施期間**

総研究期間：実施計画のjRCT公表日～2030年9月30日（6年）

（予定登録期間：2年、観察期間：登録終了後3年、解析期間：1年）

**18．研究対象者に対する説明および同意**

**18.1　説明文書および同意文書の作成**

本研究に参加する研究対象者から同意を取得するために、本研究の内容を説明する文書（説明文書）と同意の証となる文書（同意文書）を、研究計画書とは別の様式として作成する。説明文書および同意文書の作成においては、研究対象者が理解しやすいよう平易な表現となるよう配慮し、以下の説明事項を記載する。

１）本研究の名称、本研究の実施について実施医療機関の管理者の承認を受けている旨および厚生労働大臣に実施計画を届け出している旨

２）研究責任医師の氏名および職名

３）研究対象者として選定された理由

４）本研究の実施により予期される利益および不利益

５）本研究への参加を拒否することは任意である旨

６）同意の撤回に関する事項

７）本研究への参加を拒否することまたは同意を撤回することにより不利益な取扱いを受けない旨

８）本研究に関する情報公開の方法

９）研究対象者の求めに応じて、研究計画書その他の本研究の実施に関する資料を入手または閲覧できる旨およびその入手または閲覧の方法

10）研究対象者の個人情報の保護に関する事項

11）試料等の保管および廃棄の方法

12）本研究に対する利益相反管理に関する状況

13）苦情および問合せへの対応に関する体制

14）本研究の実施に係る費用に関する事項

15）他の治療法の有無および内容ならびに他の治療法により予期される利益および不利益との比較

16）本研究の実施による健康被害に対する補償および医療の提供に関する事項

17）本研究の審査意見業務を行う認定臨床研究審査委員会における審査事項その他本研究に係る認定臨床研究審査委員会に関する事項

18）その他本研究の実施に関し必要な事項

研究責任医師は、説明文書および同意文書を認定臨床研究審査委員会による承認を受けた後に厚生労働大臣に届け出る。また、研究対象者から同意取得後、研究への参加の継続について研究対象者の意思に影響を与える可能性がある情報が得られたときは、速やかに説明文書を改訂し、認定臨床研究審査委員会の承認後に厚生労働大臣に届け出る。

**18.2　同意取得（インフォームド・コンセント）**

認定臨床研究審査委員会で承認され、厚生労働大臣に届け出た説明文書および同意文書を研究対象者に渡し、同意を強制したり不当な影響を及ぼさないように文書および口頭による十分な説明を行い、研究対象者本人が同意文書に署名と日付を記入することにより、研究対象者の自由意思による同意を得る。研究対象者の同意に影響を及ぼすと考えられる有効性や安全性等の情報が得られたときや、研究対象者の同意に影響を及ぼすような実施計画等の変更が行われるときは、速やかに研究対象者に情報提供し、研究への参加を継続するか否かについて研究対象者の意思を予め確認するとともに、当該情報が反映され認定臨床研究審査委員会による承認および厚生労働大臣に届け出た説明文書および同意文書を用いて、研究対象者の再同意を得る。

**19．利益相反に関する事項**

本研究は、日本メドトロニック株式会社との研究契約に基づき、当該企業より研究機器（メドトロニック　LINQⅡ）および研究資金の提供を受けて実施する。なお、その他の研究実施に係る費用については、循環器内科学分野の研究費（運営費等）を用いる。統計解析については、研究責任医師が指名した統計解析責任者が行い、日本メドトロニック株式会社が解析に係わることはない。

本研究の実施に関与するすべての者は、実施ならびに成果に関して利益相反が生じる可能性に関し、それぞれが所属する機関における利益相反に関する規定に基づき適切にこれを管理する。

研究責任医師は、本研究に関する利益相反管理基準に基づき以下の関与の有無を確認し、その内容を実施医療機関の管理者等の確認を受けた上で利益相反管理計画を作成し、認定臨床研究審査委員会に提出する。

１）本研究に対する医薬品等製造販売業者等による研究資金等の提供その他の関与

２）本研究に従事する者（研究責任医師、研究分担医師、統計的な解析を行うことに責任を有する者等）および研究計画書に記載されている者であって、本研究を実施することによって利益を得ることが明白な者に対する本研究に用いる医薬品等の製造販売をし、またはしようとする医薬品等製造販売業者等による寄附金、原稿執筆および講演その他の業務に対する報酬の提供その他の関与

研究責任医師は、本研究の計画・実施・報告において、研究の結果および結果の解釈に影響を及ぼすような新たな利益相反状況が生じていないか本研究に従事する者に継続的に確認し、研究の実施が研究対象者の権利・利益を損ねることがないことを確認する。確認の結果、新たに利益相反状況が生じた場合にあっては、研究責任医師は利益相反管理計画を変更し、認定臨床研究審査委員会に提出する。なお、本研究の期間中は年に一度、利益相反状況について確認の上、本研究の定期報告の際に研究責任医師が認定臨床研究審査委員会に報告する。

**20．知的財産権**

本研究により得られた結果やデータは、研究責任医師（または本研究の実施医療機関）に帰属する。利用権に関しては別途契約書に定める通りとする。

また、本研究に関連して、特許権その他の知的財産権が生じた場合または生じる可能性がある場合は、研究機器の製造販売業者である日本メドトロニック株式会社に通知し、その帰属や取扱いについて協議し、決定するものとする。

**21．個人情報等の取扱い**

**21.1　個人情報の保護**

本研究で収集する研究対象者の個人情報を含むデータは、実施医療機関からEDCシステムに入力する際に個人情報を削除して、本研究用の研究対象者識別コード（研究対象者ID）を付与する。実施医療機関においては、研究対象者個人が識別できないような措置を講じ、対照表を作成し保管する。研究実施施設はEDCシステムへ匿名化対照表を提供しない。ただし、研究責任医師が指名するモニタリング担当者や臨床研究審査委員会が、本研究に関する記録を閲覧する場合がある。研究対象者は報告書の中では識別コードによってのみ特定可能とする。研究成績の学会、論文等への公表の際も、研究対象者の秘密は保全されなければならない。これらの情報の管理は、「14.4 情報の保管と廃棄」の項に従い適切に対応するほか、個人情報保護に関して実施医療機関内に別途規程や手順がある場合はその規程や手順に従い適切に対応する。

**21.2　データの二次利用**

本研究で得られたデータは改めて認定臨床研究審査委員会または倫理審査委員会等の承認を経て二次利用される可能性があり、記録の保存に記載された期間を超えて保存される可能性があるが、その場合も研究対象者の個人情報は「21.1 個人情報の保護」の項と同様の方法で保護される。

**22．研究計画書の遵守および研究計画書の変更**

**22.1　研究計画書の遵守**

研究責任医師および研究分担医師は、研究対象者の安全と人権を損なわない限り、研究計画書を遵守して本研究を実施する。

**22.2　研究計画書の変更**

１）研究責任医師は、研究計画書（説明文書および同意文書を含む）を変更する場合は、当該変更前に認定臨床研究審査委員会の審査を受け、その変更内容を実施医療機関の定める手順に従い管理者に通知する。研究責任医師または研究分担医師は、認定臨床研究審査委員会の承認または実施医療機関の管理者の承認を得る前に、変更した研究計画書（説明文書および同意文書）にて研究を実施してはならない。

２）研究計画書の変更が実施計画の変更を伴う場合には、研究責任医師は当該変更について認定臨床研究審査委員会の承認を受けた後、厚生労働大臣へ実施計画の変更届を提出しなければならない。研究責任医師または研究分担医師は、研究責任医師が厚生労働大臣へ実施計画の変更届を提出し、当該変更内容がjRCTで公表される前に、変更した研究計画書（説明文書および同意文書）にて研究を実施してはならない。

**23．不適合の管理**

１）研究責任医師は、本研究が臨床研究法、施行規則または研究計画書に適合していない状態（以下「不適合」という。）であると知ったときは、速やかに、実施医療機関の管理者に報告する。また、研究分担医師は不適合であると知ったときは、速やかに研究責任医師に報告する。

２）研究責任医師は、不適合のうち特に重大なもの（臨床研究の対象者の人権や安全性及び研究の進捗や結果の信頼性に影響を及ぼすものをいう。例えば選択・除外基準や中止基準、併用禁止療法等の不遵守等）が判明した場合においては、速やかに認定臨床研究審査委員会へ報告する。また、実施医療機関の管理者は、当該「重大な不適合」に関する対応の状況等を公表する。

**24．定期報告**

１）研究責任医師は、本研究の実施計画がjRCTで公表された日から起算して１年ごとに、当該期間満了後の2ヶ月以内に次に掲げる本研究の実施状況について、実施医療機関の管理者に報告した上で、本研究の継続の適否について意見を聴くために認定臨床研究審査委員会に報告する。

（１）本研究に参加した研究対象者の数

（２）本研究に係る疾病等の発生状況およびその後の経過

（３）本研究に係る不適合の発生状況およびその後の対応

（４）本研究の安全性および科学的妥当性についての評価

（５）本研究に対する利益相反管理に関する事項

２）研究責任医師は、認定臨床研究審査委員会が意見を述べた日から１ヶ月以内に、厚生労働大臣に以下の事項を報告する。

（１）認定臨床研究審査委員会の名称

（２）当該認定臨床研究審査委員会による当該特定臨床研究の継続の適否

（３）本研究に参加した研究対象者の数

**25．研究の中止**

研究責任医師は、本研究を中止する場合は、以下の通り対応する。

１）本研究の研究対象者に適切な措置を講ずる。

２）研究の中止日から10日以内に、研究を中止する旨を認定臨床研究審査委員会に通知するとともに、厚生労働大臣に届け出る。なお、必要に応じて研究対象者の措置に伴う研究終了時期やその方法について、認定臨床研究審査委員会の意見を聴くこと。

３）厚生労働大臣に届け出た後も本研究が終了するまでの間は、疾病等報告、定期報告等を行う他、本研究の進捗状況に関する事項の変更に該当する場合には、実施計画の変更も届け出る。

**26．研究の終了**

**26.1　総括報告書の作成**

研究責任医師は、本研究の内容に関する事項として記載した全ての評価項目に係るデータの収集を行うための期間が終了したときは、原則としてその日から１年以内に以下の事項を含めた総括報告書（臨床研究の結果等を取りまとめた文書）およびその概要を作成する。なお、研究を中止した場合であって中止届を厚生労働大臣に届け出し研究対象者の措置を終えた場合においては、中止した日または全ての評価項目に係るデータの収集を行うための期間が終了した日のいずれか遅い日から１年以内に作成する。

（１）研究対象者の背景情報（年齢、性別等）

（２）本研究のデザインに応じた進行状況に関する情報（研究対象者数の推移等）

（３）疾病等の発生状況のまとめ

（４）主要評価項目および副次評価項目のデータ解析および結果

また、当初の統計的な解析計画からの変更が発生した場合には、その変更内容等を総括報告書に記載する。

**26.2　総括報告書の届出**

１）研究責任医師は、総括報告書およびその概要を作成したときは、認定臨床研究審査委員会に意見を聴き、遅滞なく実施医療機関の管理者に提出する。

２）研究責任医師は、認定臨床研究審査委員会が総括報告書およびその概要に関する意見を述べた日から起算して１ヶ月以内に、総括報告書の概要を厚生労働大臣に以下の書類を添えて届け出る。

・最新版の研究計画書（説明文書および同意文書を含む）

３）研究責任医師は、総括報告書の概要を厚生労働大臣に提出し、jRCTで公表した時は、実施医療機関の管理者に報告する。
