# Supplementary material for: Evaluation of continuous arrhythmia monitoring using an implantable loop recorder in heart failure patients with a reduced ejection fraction: The LINQ2-HF trial rationale and protocol
Source: PLoS One. 2025 Aug 28;20(8):e0321604. doi: 10.1371/journal.pone.0321604 (PMC12393711; doi:10.1371/journal.pone.0321604)
Supplement: S3 File — (DOCX) [file pone.0321604.s003.docx]

**S3. Copy of the protocol that was approved by the ethics committee (English)**

**Title**
A Study on the Utility of Continuous Arrhythmia Monitoring Using an Implantable Loop Recorder in Patients with Heart Failure with Reduced Ejection Fraction

**Clinical Research Protocol**
(Specified Clinical Research)

Research Protocol Number: NR2024-003

Date: July 13, 2024, Version 1.0

### **List of Abbreviations**

| **Abbreviation** | **Description** |
| --- | --- |
| AF | Atrial fibrillation (心房細動) |
| BNP | Brain natriuretic peptide (脳性ナトリウム利尿ペプチド) |
| CIED | Cardiovascular implantable electronic device (植込み型心臓デバイス) |
| CRB | Certified Review Board (臨床研究審査委員会) |
| CRF | Case Report Form (症例報告書) |
| CTCAE | Common Terminology Criteria for Adverse Events (有害事象 共通用語規準) |
| EDC | Electronic Data Capture (電子的臨床検査情報収集) |
| FAS | Full analysis set (最大の解析対象集団) |
| GCP | Good Clinical Practice (医薬品の臨床試験の実施の基準に関する省令) |
| HFrEF | Heart failure with reduced ejection fraction (左室駆出率の低下した心不全) |
| ICD | Implantable cardioverter defibrillator (植込み型除細動器) |
| ILR | Implantable loop recorder (植込み型ループレコーダー) |
| IVS | Interventricular septum thickness (心室中隔厚) |
| jRCT | Japan Registry of Clinical Trials (臨床研究等提出・公開システム) |
| LAD | Left atrial diameter (左房径) |
| LVDd | Left ventricular end-diastolic diameter (左室拡張末期径) |
| LVDs | Left ventricular end-systolic diameter (左室収縮末期径) |
| LVEF | Left ventricular ejection fraction (左室駆出率) |
| LVPW | Left ventricle posterior wall thickness (左室後壁厚) |
| PMDA | Pharmaceuticals and Medical Devices Agency (医薬品医療機器総合機構) |
| PPS | Per protocol set (研究計画書に適合した対象集団) |
| SAS | Safety Analysis Set (安全性解析対象集団) |
| SGLT | Sodium glucose co-transporter (ナトリウム－グルコース共輸送体) |
| TIA | Transient ischemic attack (一過性脳虚血発作) |

### **Table of Contents**

1. Overview
2. Clinical Research Implementation Structure
3. Background of the Clinical Research
4. Purpose of the Clinical Research
5. Details of the Clinical Research
   5.1 Primary and Secondary Endpoints
   5.2 Research Design and Outline
   5.2.1 Research Design
   5.2.2 Research Outline
   5.3 Subject Registration/Allocation Method
   5.4 Overview of the Investigational Device
   5.5 Procedures for Managing the Investigational Device
   5.6 Planned Participation Period for Subjects
   5.7 Discontinuation Criteria for the Overall Study
6. Criteria for the Selection and Exclusion of Study Subjects
   6.1 Study Subjects (Target Disease)
   6.2 Inclusion Criteria
   6.3 Exclusion Criteria
7. Treatment for Study Subjects
   7.1 Implementation Procedures for the Study Treatment
   7.1.1 From Informed Consent to Registration, Evaluations at Registration
   7.1.2 ILR Implantation
   7.1.3 Remote Monitoring
   7.1.4 Outpatient Follow-up
   7.1.5 End of the Study
   7.2 Observation/Examination Items and Schedule
   7.2.1 Schedule
   7.2.2 Observation/Examination Items
   7.3 Regulations on Concomitant Medications (Therapies)
   7.3.1 Permissible/Restricted Concomitant Drugs (Therapies)
   7.3.2 Prohibited Concomitant Drugs (Therapies)
   7.4 Instructions to Study Subjects
   7.5 Post-Study Arrangements
   7.6 Discontinuation Criteria for Each Study Subject
8. Evaluation of Efficacy
   8.1 Efficacy Endpoints
   8.1.1 Primary Endpoint
   8.1.2 Secondary Endpoints
   8.2 Evaluation and Recording of Efficacy Endpoints
   8.3 Method and Timing of Analysis for Efficacy Endpoints
9. Evaluation of Safety
   9.1 Safety Endpoints
   9.2 Evaluation, Recording, and Analysis Methods for Safety Endpoints and Timing of Implementation
   9.3 Method and Timing of Analysis for Safety Endpoints
   9.4 Procedures for Collecting, Recording, and Reporting Information on Diseases, etc.
   9.4.1 Definitions of Diseases, etc.
   9.4.2 Scope and Reporting Period for Diseases, etc.
   9.4.3 Procedures for Reporting Diseases, etc.
   9.5 Observation of Study Subjects After Occurrence of Diseases, etc.
10. Statistical Analysis
    10.1 Analysis Sets
    10.1.1 Full Analysis Set (FAS)
    10.1.2 Per Protocol Set (PPS)
    10.1.3 Safety Analysis Set (SAS)
    10.2 Target Sample Size and Rationale for the Setting
    10.3 Handling of Cases
    10.4 Handling of Data
    10.5 Statistical Analysis Items and Analysis Plan
    10.5.1 Summary of Baseline Characteristics
    10.5.2 Analysis of the Primary Endpoint
    10.5.3 Analysis of the Secondary Endpoints
    10.5.4 Analysis of the Safety Endpoints
    10.5.5 Interim Analysis
    10.6 Final Analysis
11. Access to Source Documents, Etc.
12. Quality Control and Quality Assurance
    12.1 Monitoring
    12.2 Auditing
13. Ethical Considerations
    13.1 Compliance with Laws and Regulations
    13.2 Expected Benefits, Burdens, and Disadvantages
    13.3 Handling of Genetic Characteristics and Incidental Findings
14. Handling and Storage of Records (Including Data) and Samples
    14.1 Methods of Data Collection
    14.2 Preservation of Records
    14.3 Storage of Samples, Etc.
    14.4 Storage and Disposal of Information
15. Financial Compensation and Insurance
    15.1 Payment of Money (Cost-Burden on Study Subjects)
    15.2 Insurance Arrangements
16. Publication of Information
17. Implementation Period
18. Explanation and Consent for Study Subjects
    18.1 Preparation of Explanatory and Consent Documents
    18.2 Obtaining Informed Consent
19. Conflict of Interest
20. Intellectual Property Rights
21. Handling of Personal Information, Etc.
    21.1 Protection of Personal Information
    21.2 Secondary Use of Data
22. Adherence to and Amendments of the Research Protocol
    22.1 Adherence to the Research Protocol
    22.2 Amendments of the Research Protocol
23. Management of Noncompliance
24. Periodic Reporting
25. Discontinuation of the Study
26. Completion of the Study
    26.1 Preparation of the Final Clinical Study Report
    26.2 Submission of the Final Clinical Study Report

### **1. Overview**

**Research Title**
A Study on the Utility of Continuous Arrhythmia Monitoring Using an Implantable Loop Recorder in Patients with Heart Failure with Reduced Ejection Fraction

**Purpose of the Study**
To investigate the clinical utility of continuous heart rhythm monitoring using an implantable loop recorder (ILR) in detecting asymptomatic arrhythmias in patients with heart failure with reduced ejection fraction (HFrEF)—patients who have not been diagnosed with atrial fibrillation (AF).

**Research Design**
Single-center, open-label, non-controlled study

**Nature of the Study**
Exploratory research

**Investigational Medical Devices**

- **General name**: Implantable data recorder for electrocardiograms
  **Product name**: Medtronic LINQ II
  **Classification**: Highly controlled medical device (Class IV)
  **Manufacturer/Distributor**: Medtronic Japan

Note: Includes “MyCareLink Relay Home Communicator” as a component.

- **General name**: Program for management of implantable active devices
  **Product name**: Medtronic Reveal LINQ Mobile Manager
  **Classification**: Highly controlled medical device (Class III)
  **Manufacturer/Distributor**: Medtronic Japan
- **General name**: Programmer for implantable active devices
  **Product name**: Medtronic 24967 Patient Connector
  **Classification**: Highly controlled medical device (Class III)
  **Manufacturer/Distributor**: Medtronic Japan

**Subjects**
Patients with HFrEF who have not been diagnosed with AF

**Inclusion Criteria**
All of the following conditions must be met:

1. Patients with HFrEF (left ventricular ejection fraction ≤ 40%)
2. Patients who are undergoing appropriate pharmacotherapy for HFrEF and are currently in outpatient care or are able to attend outpatient care
3. Patients with a CHADS2 score ≥ 1
4. Age ≥ 20 at the time of consent
5. Patients who provide written informed consent

**Exclusion Criteria**
Any of the following:

1. Patients who already have an implanted cardiovascular electronic device (CIED) capable of detecting supraventricular arrhythmias (i.e., a permanent pacemaker, ICD, or ILR)
2. Patients diagnosed with AF at the time of consent
3. Patients whose life expectancy is considered to be within 1 year
4. Patients in an immunocompromised state
5. Patients with an active infection
6. Patients whose subcutaneous tissue is too thin at the implantation site, making safe ILR implantation difficult
7. Any patient deemed inappropriate for this study by the principal or sub-investigator

**Endpoints**

- **Primary Endpoint**
  Proportion of patients in whom AF or non-sustained or sustained ventricular tachycardia is detected

Defined as the proportion of patients (in the analysis population) who exhibit the following on ILR:

- - AF of ≥6 minutes duration
  - Non-sustained VT: rate >150 bpm, ≥16 consecutive beats, ≤30 seconds
  - Sustained VT: rate >150 bpm, ≥30 seconds
- **Secondary Endpoints**
  - Proportion of patients with pauses ≥4.5 seconds
  - Proportion of patients with bradycardia <30 bpm
  - Proportion of patients with high-grade AV block
  - Proportion of patients who received a permanent pacemaker, ICD, catheter ablation, or antiarrhythmic drug therapy
  - All-cause mortality rate
  - Cardiovascular mortality rate
  - Proportion of patients requiring hospitalization due to cardiovascular disease or heart failure
  - Proportion of patients who experienced stroke
- **Safety Endpoints**
  - Incidence of adverse events related to ILR implantation
  - Incidence of device malfunctions

**Protocol Treatment**
After obtaining written informed consent, the ILR will be implanted subcutaneously in the left anterior chest area under local anesthesia by the principal or sub-investigator. Post-implantation, remote monitoring (using Medtronic CareLink) will be performed every 6 months to assess the arrhythmic events specified in Section 5.1.

For the primary endpoint and secondary endpoints 1–3, if an arrhythmic event is detected via remote monitoring, an alert email will be sent or forwarded to an evaluation physician who is separate from the outpatient attending physician. The evaluation physician will review the ECG stored on the server and make a diagnosis (determine whether an arrhythmic event occurred). If the physician judges that immediate communication with the outpatient physician is medically necessary, the monitoring results will be reported to the outpatient physician as needed. If the patient experiences symptoms suggestive of arrhythmia, the ILR data may be checked at any time.

Patients will continue outpatient visits every 1–3 months, as before ILR implantation. During these visits, the presence or absence of events related to secondary endpoints 4–8 will be verified, along with other clinical progress.

**Target Sample Size**
35 subjects

**Study Period**
Overall study period: From the date of jRCT publication of the implementation plan to September 30, 2030 (6 years)

- Planned enrollment period: 2 years
- Observation period: 3 years after enrollment ends
- Analysis period: 1 year

**Number of Study Sites**
Single-center (Tokyo Medical and Dental University Hospital)

**2.Clinical Research Implementation Structure**

| **Role** | **Name** | **Title** | **Affiliation/Department** |
| --- | --- | --- | --- |
| Principal Investigator | Shinsuke Miyazaki | Associate Professor (Endowed) | Tokyo Medical and Dental University Hospital, Dept. of Cardiology |
| Data Management Supervisor | Taeko Murakami | URA | Tokyo Medical and Dental University, Health Sci. R&D Center |
| Monitoring Supervisor | Junko Taniguchi | URA | Tokyo Medical and Dental University, Health Sci. R&D Center |
| Monitoring Supervisor | Yukisetsu Kuwamoto | URA | Tokyo Medical and Dental University, Health Sci. R&D Center |
| Biostatistics Supervisor | Ryoichi Hanazawa | Special Appointed Assistant Prof. | TMDU, Health Sci. R&D Ctr. / Dept. of Clinical Statistics |
| Biostatistics Researcher | Akihiro Hirakawa | Professor | TMDU, Dept. of Clinical Statistics |
| Biostatistics Researcher | Hiroyuki Sato | Assistant Prof. | TMDU, Dept. of Clinical Statistics |
| Biostatistics Researcher | Ryo Kitabayashi | Special Appointed Assistant Prof. | TMDU, Health Sci. R&D Ctr. / Dept. of Clinical Statistics |
| R&D Planning Support | Ayako Sakai | URA | TMDU, Health Sci. R&D Center |
| R&D Planning Support | Megumi Ishiguro | Associate Professor | (Same) |
| Remote Monitoring Evaluation Physicians | Ryo Tateishi | Assistant Prof. | TMDU Hospital, Dept. of Cardiology |
|  | Kazuya Yamao | Assistant Prof. | (Same) |

(Address: 1-5-45 Yushima, Bunkyo-ku, Tokyo)

### **3. Background of the Clinical Research**

In Japan’s statistics of causes of death, heart disease ranks second; among these, heart failure accounts for the largest proportion. With an aging society, the number of heart failure patients continues to increase, projected to reach 1.2 million by 2020. About half of these patients have reduced left ventricular ejection fraction, and the 2017 revised “Guidelines for Diagnosis and Treatment of Acute and Chronic Heart Failure” define HFrEF (Heart Failure with reduced Ejection Fraction) as an LVEF <40%.

Atrial fibrillation (AF) is the most frequently coexisting arrhythmia in heart failure, and its prevalence increases in more severe heart failure cases, serving as a trigger for both exacerbation of heart failure and thromboembolism. If detected at an early stage, ablation or antiarrhythmic drug therapy can be used for rhythm control; in chronic cases, the arrhythmia may be managed by rate control with medication. Ventricular arrhythmias are also common in HFrEF and can lead to sudden cardiac death. High-risk cases may require antiarrhythmic drug therapy or implantation of an implantable cardioverter defibrillator (ICD).

Since arrhythmias typically occur intermittently and often without symptoms, diagnosing them in regular outpatient care can be difficult. It is known that the diagnostic accuracy for arrhythmias depends on the length of time ECG is evaluated (monitoring period), and implantable loop recorders (ILRs)—implanted subcutaneously in the left anterior chest—allow continuous ECG monitoring and thus provide the highest accuracy. The “Medtronic LINQ II” device we will use in this study is small and light (45 × 8 × 4.2 mm, 4 g), and its monitoring data can be checked by healthcare providers at any time via a remote monitoring system. While it is an already-approved device in Japan, domestic approval is limited to (1) patients with unexplained syncope and (2) patients with unexplained cerebral infarction. By contrast, it has broader indications in the U.S. and Europe, including use for AF monitoring. For instance, a large clinical trial by Sanna et al., involving 441 patients with cryptogenic stroke in whom AF was not detected by conventional means, randomized them into ILR implantation vs. conventional monitoring groups for 6 months, demonstrating a significantly higher detection rate of AF in the ILR group (8.9% vs. 1.4%).

For HFrEF patients (who are at high risk of developing AF and other arrhythmias), continuous ECG monitoring by ILR may facilitate the earlier detection of arrhythmias and earlier therapeutic intervention, potentially improving mortality.

Since there are no published data on ILR-based continuous ECG monitoring in the target population of this study (patients with HFrEF), the exact incidence of newly identified arrhythmias remains unclear. This study is exploratory, enrolling up to 35 patients who are likely eligible for ILR implantation over 2 years, to examine the exploratory utility of ILR for HFrEF. Patients may benefit from earlier detection and intervention for arrhythmias. Potential disadvantages include skin-related complications (infection, erosion, bleeding, etc.) at the implantation site. Early overseas reports in patients receiving ILRs for unexplained syncope, unexplained stroke, or AF monitoring indicated a complication rate of about 1.5%, with about half resolving conservatively and the other half requiring device explant. We will follow subjects for one year (the main analysis time point) and continue observation for up to three years, matching the battery life of the device. Should promising results be obtained, we will consider, in collaboration with the manufacturer, the possibility of conducting a subsequent clinical trial aimed at expanding indications to use ILRs for HF patients.

1. Okura Y, et al. Impending epidemic: future projection of heart failure in Japan to the year 2055. Circ J 2008; 72: 489-491.
2. Guidelines for Diagnosis and Treatment of Acute and Chronic Heart Failure （JCS 2017/JHFS 2017）
3. JCS/JHRS 2019 Guideline on Non-Pharmacotherapy of Cardiac Arrhythmias
4. Aguilar M, et al. Influence of Monitoring Strategy on Assessment of Ablation Success and Postablation Atrial Fibrillation Burden Assessment: Implications for Practice and Clinical Trial Design. Circulation. 2022;145:21-30.
5. Sanna T, et al. Cryptogenic stroke and underlying atrial fibrillation. N Engl J Med. 2014;370:2478-86.
6. Mittal S, et al. Safety Profile of a Miniaturized Insertable Cardiac Monitor: Results from Two Prospective Trials. Pacing Clin Electrophysiol. 2015;38:1464-9.

### **4. Purpose of the Clinical Research**

To investigate the clinical utility of continuous heart rhythm monitoring using ILRs in detecting asymptomatic arrhythmias among HFrEF patients not diagnosed with AF.

### **5. Details of the Clinical Research**

#### 5.1 Primary and Secondary Endpoints

#### (See “8. Evaluation of Efficacy” for details.) The primary analysis will use data at 1 year post-implantation; after all patients have completed the 3-year follow-up, a final analysis will be performed.

- **Primary Endpoint**
  Proportion of patients in whom AF or non-sustained or sustained VT is detected

Definition: Among the analysis population, the percentage of patients with any of:

- - AF lasting ≥6 minutes
  - Non-sustained VT: heart rate >150 bpm, ≥16 consecutive beats, ≤30 s
  - Sustained VT: heart rate >150 bpm, lasting ≥30 s

Rationale: These arrhythmias would clinically warrant consideration of intervention (pharmacological, ablation, or device-based).

- **Secondary Endpoints**
  - Proportion of patients with pauses ≥4.5 seconds
  - Proportion of patients with bradycardia <30 bpm
  - Proportion of patients with high-grade AV block
  - Proportion of patients receiving permanent pacemakers, ICDs, catheter ablation, or antiarrhythmic drug therapy
  - All-cause mortality
  - Cardiovascular mortality
  - Proportion of patients requiring hospitalization for cardiovascular disease or heart failure
  - Proportion of patients experiencing stroke

Rationale:

- - (1)–(3): They may require consideration of therapeutic intervention.
  - (4): To evaluate the effect of continuous ECG monitoring by ILR on treatment decisions.
  - (5)–(8): To evaluate the relationship between ILR findings and clinical outcomes.
- **Safety Evaluation Items**
  - Incidence of adverse events related to ILR implantation
  - Incidence of malfunctions

(Rationale: General safety assessment items.)

#### 5.2 Research Design and Outline

##### 5.2.1 Research Design

1. Nature of the study: exploratory
2. Randomization: single-arm
3. Blinding: open-label
4. Control: none
5. Allocation: none

##### 5.2.2 Research Outline

Remote monitoring via the CareLink system (Medtronic) will be conducted every 6 months for 3 years post-implantation. Outpatient follow-up visits every 1–3 months will continue as before.

#### 5.3 Subject Registration and Allocation Method

1. **Registration Method**
   The principal or sub-investigator will confirm that the patient meets all inclusion and no exclusion criteria after obtaining written informed consent. Then, those engaged in this clinical research will enter the patient data into the EDC system to register the subject.
2. **Allocation Method**
   There is no group allocation in this single-arm study.

#### 5.4 Overview of the Investigational Device

We will use the following devices. The “Medtronic Reveal LINQ Mobile Manager” and “Medtronic 24967 Patient Connector” are already available at the study institution.

- **General Name**: Implantable ECG Data Recorder
  **Product Name**: Medtronic LINQ II
  **Class**: Highly controlled medical device (Class IV)
  **Intended Use/Effect**: For patients with unexplained syncope/palpitations, or for monitoring AF in cryptogenic stroke patients, by continuous subcutaneous ECG recording.
  **Approval Number**: 30300BZX00278000 (October 2021)
  **Manufacturer**: Medtronic Japan
- **General Name**: Program for management of implantable active devices
  **Product Name**: Medtronic Reveal LINQ Mobile Manager
  **Class**: Class III
  **Approval Number**: 22800BZX00305000 (August 2016)
  **Manufacturer**: Medtronic Japan
- **General Name**: Programmer for implantable active devices
  **Product Name**: Medtronic 24967 Patient Connector
  **Class**: Class III
  **Approval Number**: 30100BZX00034000 (June 2019)
  **Manufacturer**: Medtronic Japan

Because AF monitoring is not an approved indication in Japan (currently limited to unexplained syncope and cryptogenic stroke), its use for AF monitoring in HFrEF is off-label domestically, although widely used overseas for AF detection.

**Usage Summary**: LINQ II is a small, lightweight ECG monitoring device (45×8×4.2 mm, 4 g). It is implanted about 8 mm under the skin in the left anterior chest using a specialized kit, and configured for remote monitoring with the dedicated connector/programmer. ECG data are periodically transmitted to the server, allowing the medical staff to review the patient’s ECG as needed.

#### 5.5 Procedures for Managing the Investigational Device

The devices are supplied by Medtronic Japan to the principal investigator and stored at the designated location in TMDU Hospital. Storage conditions and usage deadlines adhere to the package insert. The principal investigator is responsible for device management.

#### 5.6 Planned Participation Period for Subjects

Approximately 3 years after providing consent. If an adverse event is suspected to be related to the study beyond that period, follow-up will continue until it is deemed resolved.

#### 5.7 Discontinuation Criteria for the Overall Study

The principal investigator will consider whether to continue the study if any of the following occurs:

- Discovery of critical information impacting device quality, performance, or safety
- Difficulty in enrolling enough subjects
- Early achievement of study objectives
- Instructions to modify the protocol from the Certified Review Board that cannot be accepted
- The CRB discontinues the study
- Serious or ongoing violations of the Clinical Trials Act, its enforcement regulations, or this protocol

### **6. Criteria for Selection and Exclusion of Study Subjects**

#### 6.1 Study Subjects (Target Disease)

Patients with HFrEF who are not diagnosed with AF

#### 6.2 Inclusion Criteria

All conditions must be met:

1. HFrEF (LVEF ≤40%)
2. Receiving appropriate pharmacotherapy for HFrEF; currently attending outpatient or able to do so
3. CHADS2 score ≥1
4. Age ≥20 at time of consent
5. Written informed consent

#### 6.3 Exclusion Criteria

Any of the following:

1. Already implanted with a CIED capable of detecting supraventricular arrhythmias
2. Diagnosed with AF at time of consent
3. Life expectancy <1 year
4. Immunocompromised
5. Active infection
6. Subcutaneous tissue too thin at implant site for safe ILR placement
7. Otherwise deemed inappropriate

### **7. Treatment for Study Subjects**

#### 7.1 Implementation Procedures for the Study Treatment

##### 7.1.1 From Informed Consent to Registration, Evaluations at Registration

- The principal/sub-investigator will confirm eligibility and explain the study, obtaining written informed consent.
- After consent, baseline data and relevant tests will be done (see “7.2”).
- Registration is completed by entering subject data into the EDC.
- If required tests performed within 30 days before registration are available, those can be used.
- Baseline ECG: the heart rhythm is confirmed on a monitor ECG at ILR implantation.

##### 7.1.2 ILR Implantation

- Within 30 days after registration, the ILR is implanted (usually outpatient basis, possibly inpatient if necessary). If it cannot be done within 30 days, protocol treatment is discontinued.
- Implantation steps (outline):
  1. Implant site: 4th intercostal space in the left anterior chest.
  2. Local disinfection with povidone-iodine, local anesthesia, small incision, creation of a subcutaneous pocket (~8 mm depth).
  3. Insert the ILR device using the supplied introducer.
  4. Confirm adequate ECG signal via the programmer (Reveal LINQ Mobile Manager) before closing.
  5. No prophylactic antibiotics are used.
  6. The device is programmed for continuous monitoring (remote monitoring) settings.

##### 7.1.3 Remote Monitoring

- The CareLink system (Medtronic) transmits data from a home-based communicator (“MyCareLink Relay”) to a dedicated server. Physicians can access the data at any time.
- Every 6 months for 3 years, a separate “evaluation physician” (not the outpatient physician) checks the arrhythmia event data from the ILR. The results are recorded in the CRF.
- If the ILR records an arrhythmia event, an alert email is sent to the evaluation physician, who then reviews the ECG on the server to confirm. If urgent, the evaluation physician notifies the outpatient physician immediately. If the patient reports symptoms, the ILR data can be reviewed at any time.

##### 7.1.4 Outpatient Follow-up

- The patient continues outpatient visits every 1–3 months. During those visits, events related to secondary endpoints 4–8 and the subject’s clinical status are confirmed.
- Additional tests may be performed as necessary for standard care.
- No extra examinations are performed solely for this study.
- If the subject moves away, it may still be possible to continue in the study if certain conditions are met (e.g., medical records can be obtained from a local hospital, the subject agrees to return for ILR explantation unless physically impossible, etc.).

##### 7.1.5 End of the Study

- At 3 years post-implantation, the subject completes the protocol observation period, and the ILR is explanted.
- If an adverse event or device malfunction (e.g., battery depletion) necessitates explantation, the study observation ends at that time.
- If a new device (e.g., a permanent pacemaker) is implanted, rendering ILR monitoring moot, ILR is explanted, and observation ends.
- If follow-up is no longer feasible, the ILR is explanted, and observation ends.
- Explantation is generally performed on an outpatient basis with local anesthesia. The removed ILR is discarded per institutional procedures.
- The MyCareLink Relay Home Communicator is discarded as non-burnable trash.

#### 7.2 Observation/Examination Items and Schedule

##### 7.2.1 Schedule

| **Observation Items** | **Pre-Implant** | **At Implant** | **Remote Monitoring After Implant** |
| --- | --- | --- | --- |
| Informed Consent (IC) | ● |  |  |
| Registration | ● |  |  |
| Subject Background Info | ● |  |  |
| ILR Implantation |  | ● |  |
| Remote Monitoring: arrhythmia events¹ |  |  | 6/12/18/24/30/36 months ±4 weeks |
| 12-lead ECG² | ● | ● |  |
| Echo (LAD, LVDd, LVDs, LVEF, etc.) | ● |  |  |
| Blood test (BNP) | ● |  |  |
| Chest X-ray | ● |  |  |
| Clinical course (every 1–3 months) |  |  | (Ongoing in standard outpatient care) |
| Adverse events, device malfunctions | (Monitored throughout the study) |  |  |

¹ ILR data are evaluated at 6-month intervals (±4 weeks).
² Baseline ECG: can be a monitor ECG at implant.

##### 7.2.2 Observation/Examination Items

1. **Informed Consent**
   Date of written informed consent
2. **Subject Background**
   - **Basic info**: DOB, age, sex, height, weight, BMI
   - **Comorbidities**: HF, hypertension, diabetes, stroke/TIA (for CHADS2), vascular disease (for CHA2DS2-VASc)
   - **Dialysis**: presence or absence
   - **Past cardiac surgery**: type, date
   - **Hospitalization history for HF**: presence, number of admissions, most recent admission date
   - **Current medications**: antiplatelet/anticoagulants, diuretics, beta-blockers, RAA inhibitors, MRAs, SGLT2 inhibitors, other HF therapies, antiarrhythmics
3. **Pre-implantation (Registration) Tests**
   - **Echocardiogram**: LAD, LVDd, LVDs, LVEF, IVS, LVPW, etc.
   - **Blood test**: BNP
   - **ECG**: 12-lead ECG to confirm rhythm (sinus, AF/flutter, paced, etc.)
   - **Chest X-ray**: cardio-thoracic ratio (CTR)
4. **At Implantation**
   - **ECG**: Monitor ECG (or 12-lead if possible) to confirm rhythm
5. **ILR Remote Monitoring**
   - Date of evaluation, evaluator
   - **AF ≥6 minutes**: presence/absence, date/time, number of events, total duration, etc.
   - **Non-sustained VT** (HR >150, ≥16 beats, ≤30 s)
   - **Sustained VT** (HR >150, ≥30 s)
   - **Pauses ≥4.5 s**
   - **Bradycardia <30 bpm**
   - **High-grade AV block**
6. **Clinical Course**
   - Mortality (date, CV or non-CV, details)
   - CV or HF hospitalizations (date, details)
   - Stroke (date, details)
   - Implantation of a permanent pacemaker, ICD therapy, catheter ablation, AAD therapy (dates, details)
7. **Adverse Events**
   - Onset date, resolution date, severity (Grade), treatment, outcome, seriousness, relationship to ILR, whether ILR explanted, etc.
8. **Device Malfunctions**
   - Malfunction name, onset/end date, presence of adverse event, whether ILR explanted, etc.

#### 7.3 Regulations on Concomitant Medications (Therapies)

##### 7.3.1 Permissible/Restricted

MRI scans should follow the device labeling conditions.

##### 7.3.2 Prohibited

- Diathermy (radiofrequency, shortwave, microwave)
- Permanent pacemaker implantation
- ICD implantation

#### 7.4 Instructions to Study Subjects

Before starting the study, the principal/sub-investigator instructs subjects to:

1. Inform any other doctors/hospitals that they are participating in this study and ideally consult the study physician in advance if other treatment is needed.
2. Report any health concerns promptly.
3. Notify their physician in advance if MRI is needed.
4. Avoid the use of high-frequency, shortwave, or microwave diathermy devices.
5. Follow the physician’s guidance regarding diet, exercise, etc.
6. Refrain from posting study-related information on social media, etc.
7. Notify the investigator immediately if pregnancy occurs.
8. Adhere to prohibited/restricted drug/therapy guidelines.

#### 7.5 Post-Study Arrangements

After study completion, subjects will receive the best possible prevention, diagnosis, or treatment options informed by the study results. If a subject wishes to continue ILR monitoring beyond the 3-year protocol period, the original ILR will be explanted, and a new one may be implanted under self-pay.

#### 7.6 Discontinuation Criteria for Each Study Subject

1. Subject withdraws consent.
2. ILR cannot be implanted within 30 days of registration.
3. Study continuation deemed difficult by PI or sub-investigator due to worsening primary disease or severe AEs.
4. ILR explantation due to AE or malfunction.
5. Need for another CIED (e.g., permanent pacemaker), leading to ILR explant.
6. Follow-up no longer feasible due to relocation, etc., leading to explant.
7. Prohibited therapy is required or has been administered.
8. Serious deviations from the Clinical Trials Act or from the protocol.
9. Inability to adhere to the protocol.
10. Pregnancy.
11. Entire study is discontinued.
12. Other reasons deemed appropriate by the investigator.

If any discontinuation occurs, reasons and details are documented, and the subject is followed clinically as appropriate.

### **8. Evaluation of Efficacy**

#### 8.1 Efficacy Endpoints

##### 8.1.1 Primary Endpoint

Proportion of patients in whom AF or non-sustained or sustained VT is detected

##### 8.1.2 Secondary Endpoints

1. Proportion of patients with pauses ≥4.5 s
2. Proportion of patients with bradycardia <30 bpm
3. Proportion of patients with high-grade AV block
4. Proportion of patients receiving pacemaker, ICD, ablation, or antiarrhythmic therapy
5. All-cause mortality
6. Cardiovascular mortality
7. Proportion with hospitalizations for CV disease/HF
8. Proportion who experienced stroke

#### 8.2 Evaluation and Recording of Efficacy Endpoints

The main analysis uses 1-year data; after all have reached 3 years, a final analysis is done.

#### 8.3 Method and Timing of Analysis for Efficacy Endpoints

See Section 10 for statistical methods.

**9. Evaluation of Safety**

**9.1 Safety Endpoints**

The following safety endpoints will be assessed:

1. **Incidence of adverse events (AEs) related to ILR implantation:**
   - This includes any complications arising from the device implantation procedure, such as infection, erosion, bleeding, or device migration.
   - AEs will be classified based on their severity and relationship to the investigational device.
2. **Incidence of device malfunctions:**
   - Malfunctions include battery failure, data transmission issues, signal loss, and other device-related failures.
   - Malfunctions that lead to clinical consequences (e.g., an inability to detect arrhythmias) will be documented separately.

An **adverse event (AE)** is defined as any undesirable experience occurring in a subject, whether related to the study intervention or not. **A serious adverse event (SAE)** includes events that result in hospitalization, life-threatening conditions, or significant disability.

**9.2 Evaluation, Recording, and Analysis of Safety Endpoints**

1. **Evaluation Period:** From the date of ILR implantation until three years post-implantation.
2. **Data Collection:**
   - All AEs and device malfunctions will be recorded in the case report form (CRF).
   - Events will be categorized based on severity, expectedness, and causality.
3. **Reporting:**
   - Serious AEs will be reported within 24 hours to the Certified Review Board (CRB) and regulatory authorities as required.
   - Routine AE reports will be submitted periodically.

**9.3 Statistical Analysis of Safety Endpoints**

- The frequency and incidence rate of AEs and device malfunctions will be calculated.
- Kaplan-Meier survival analysis may be conducted for time-to-event data.
- Subgroup analysis will be performed based on subject demographics, comorbidities, and treatment history.

**9.4 Reporting of Diseases, Adverse Events, and Device Malfunctions**

1. **Definitions:**
   - Disease events include arrhythmias detected by ILR and other cardiovascular conditions.
   - AEs and device malfunctions will be categorized according to international clinical research guidelines.
2. **Reporting Timeline:**
   - SAE: Immediate reporting within 24 hours.
   - Other AEs: Monthly reports.
3. **Follow-up Actions:**
   - Subjects experiencing AEs will receive appropriate medical treatment and continued monitoring.
   - Device malfunctions will be addressed through technical support and, if necessary, device replacement.

**10. Statistical Analysis**

**10.1 Analysis Sets**

1. **Full Analysis Set (FAS):** Includes all enrolled subjects who received an ILR.
2. **Per Protocol Set (PPS):** Includes subjects who strictly adhered to the study protocol without major deviations.
3. **Safety Analysis Set (SAS):** Includes all subjects implanted with an ILR for safety evaluations.

**10.2 Target Sample Size**

- **Planned Enrollment:** 35 subjects
- **Rationale:**
  - Based on prior studies on ILRs, a sample size of 35 subjects is deemed sufficient to evaluate feasibility and detect major arrhythmic events.
  - Power analysis will be conducted retrospectively.

**10.3 Handling of Cases**

- Subjects who withdraw before ILR implantation will be excluded from analysis.
- Subjects lost to follow-up will be included in the last-observation-carried-forward (LOCF) analysis.

**10.4 Data Handling and Confidentiality**

- Data will be stored in a secured electronic data capture (EDC) system.
- Personal identifiers will be replaced with unique study IDs.
- Data retention period: Minimum of 10 years post-study completion.

**10.5 Statistical Analysis Plan**

1. **Baseline Characteristics:** Descriptive statistics for demographic and clinical characteristics.
2. **Primary Endpoint Analysis:**
   - Incidence of AF and ventricular tachycardia will be analyzed using proportion estimates.
   - Confidence intervals (95% CI) will be reported.
3. **Secondary Endpoint Analysis:**
   - Kaplan-Meier survival curves for time-to-event analyses.
   - Cox regression models for risk factor assessment.
4. **Safety Analysis:**
   - AE incidence and severity will be analyzed descriptively.

**10.6 Final Analysis**

- Conducted after all subjects complete the three-year follow-up.
- The study database will be locked, and results will be compiled into a final report.

**11. Items Related to Viewing of Original Materials**

In this study, “original materials” refers to medical records, various test data, records of prescriptions, etc.

Both the Principal Investigator and the administrator of each participating medical institution will agree to accept any monitoring and auditing related to this study, as well as investigations by the Certified Clinical Research Review Board and regulatory authorities. At such times, they guarantee that all relevant materials (including original materials) pertaining to this study will be made available for direct inspection.

For data documented in the case report form (CRF) which are not listed in the medical records, the following items will be treated as the original data within the CRF:

1. The purpose for the use of concomitant drugs (or therapies) and the purpose for conducting concomitant therapies
2. The severity, grade, outcome, date of outcome, and causal relationship with the investigational device for adverse events (or diseases, etc.), along with the reasons for determining the causality to the investigational device
3. The date of discontinuation, the reason for discontinuation, the adverse event or malfunction leading to discontinuation, and the subsequent course and results of follow-up after discontinuation
4. Comments by the Principal Investigator (or Sub-Investigator)

**12. Quality Control and Quality Assurance**

### 12.1 Monitoring

The Principal Investigator will develop a monitoring procedure manual for the purpose of quality control of this study and obtain review and approval from the Certified Clinical Research Review Board together with the study protocol. The Principal Investigator will also appoint a monitor responsible for carrying out the monitoring. The monitor will follow the monitoring procedure manual to verify, throughout the duration of the study, that this study is being conducted in compliance with the latest version of the study protocol and regulatory requirements (the Clinical Research Act, its enforcement regulations, etc.), and will report the findings to the Principal Investigator in accordance with the procedure manual. The monitor must not disclose any personal information of the study participants obtained through monitoring.

### 12.2 Auditing

Auditing of this study will be conducted by the Health Science R&D Center of Tokyo Medical and Dental University.

In order to ensure that all aspects of the study, as well as the creation, recording, and reporting of data, are conducted in compliance with the Clinical Research Act and its related regulations (including the enforcement regulations), the approved study protocol, and relevant procedural documents, the Principal Investigator will have an independent person perform the audit.

Specific methods and details of the audit will follow an auditing procedure manual established separately.

**13. Ethical Considerations**

### 13.1 Compliance with Laws and Regulations

All researchers involved in this study will conduct the trial in accordance with the “Declaration of Helsinki” (Japanese Medical Association translation)1) and the “Clinical Research Act” (Act No. 16 of 2017)2) and its enforcement regulations, as well as relevant notifications.

1. <http://dl.med.or.jp/dl-med/wma/helsinki2013j.pdf>
2. <http://www.mhlw.go.jp/stf/seisakunitsuite/bunya/0000163417.html>

### 13.2 Anticipated Benefits, Burdens, and Disadvantages

1. **Anticipated Benefits**
   Participation in this study will not result in any direct benefits to participants. However, the study findings could potentially contribute to the advancement of medical care in the future.
2. **Anticipated Disadvantages**
   The investigational medical device “Medtronic LINQ II” is a Class IV highly controlled medical device approved in Japan. Its use in this study population will be off-label; however, since it will be used in accordance with the instructions for use, given that it is a device for measuring electrocardiograms and is implanted subcutaneously, the burden on the study participants is considered limited. The likelihood of severe physical risk stemming from this study is deemed low.
   According to the instructions for use, device malfunctions such as early battery depletion, sensing failure, and data collection failure (frequency unknown) have been reported. There have also been reports of adverse events (frequency unknown) such as device extrusion, hematoma, bleeding, etc. Other unknown or known side effects or malfunctions may occur. If any side effects or malfunctions are observed, study personnel will administer appropriate care and provide the best possible treatment. The Principal Investigator will collect safety information and take appropriate actions, such as amending the study protocol if necessary.
3. **Anticipated Burdens**
   The number of clinic visits, tests, and the time required for examinations and tests in this study are roughly the same as those in standard medical care. There is no cost to participants for using the investigational device in this study (see “15. Payment of Money and Compensation” for details).
4. **Measures to Minimize Risks**
   This study targets outpatients as participants, and the likelihood of significant physical risk resulting from the study is considered low. Nevertheless, at each visit, the Principal Investigator (or Sub-Investigator) will be attentive to the participant’s condition and strive for early detection of any adverse events or malfunctions.

### 13.3 Handling of Genetic or Incidental Findings Related to Study Participants

This study does not involve any tests or analyses that would yield critical information about the participants’ health or genetic characteristics.

**14. Handling and Storage of Records (Including Data) and Samples**

### 14.1 Method of Data Collection

For this study, research data will be entered and managed in the electronic data capture (EDC) system “eACReSS.” The entered information will be stored on the eACReSS server at Tokyo Medical and Dental University.

Note: “eACReSS” is a dedicated clinical research data management system developed under the University Hospital Clinical Trial Alliance project. This alliance involves eight national universities in the Kanto-Koshinetsu area (the University of Tokyo, Chiba University, Tokyo Medical and Dental University, the University of Tsukuba, Gunma University, Niigata University, Shinshu University, and the University of Yamanashi).

1. **Storage location:** Inside the Tokyo Medical and Dental University “eACReSS” server
2. **Storage supervisor:** Taeko Murakami (Health Science R&D Center, Tokyo Medical and Dental University)
3. **Storage period:** 10 years after the completion of the study
4. **Destruction method:** Data will be destroyed in a way that makes restoration impossible.
5. **Possibility of secondary use:** □ None ■ Yes (see “21.2 Secondary Use of Data”)

### 14.2 Retention of Records

1. The records that must be retained for this study include the following:
   (1) Date and location where the investigational medical product, etc. was used for each participant
   (2) Items identifying the study participants
   (3) Information pertaining to treatment and tests administered to the study participants
   (4) Information regarding participation in this study
   (5) Other items necessary for conducting this study
2. The Principal Investigator must keep, for 10 years from the day this study is completed, all records that must be retained for this study together with the following documents:
   (1) Study protocol, implementation plan, documents related to explanation and consent of participants, the final report, and any other documents created by the Principal Investigator under the Clinical Research Act and its enforcement regulations, or copies of such documents
   (2) Documents pertaining to review opinions from the Certified Clinical Research Review Board
   (3) Documents related to monitoring and auditing (if auditing is conducted)
   (4) Original materials, etc.
   (5) Contracts related to the implementation of this study (excluding those contracts concluded with marketing authorization holders of pharmaceutical products, etc., or their special related parties as stipulated in Article 32 of the Clinical Research Act)
   (6) Documents describing the overview of the investigational medical product, etc. used in this study, as well as documents related to the manufacturing, acquisition, and disposal of these products
   (7) Other documents necessary for conducting this study
3. If any corrections are made to the items (1)–(5) in the retained records for this study, the name of the person making the correction and the date the correction was made must be documented, and the corrected record must be retained together with that documentation.

### 14.3 Storage of Samples

In this study, there are no plans to collect, use, or store specimens (blood, tissues, cells, bodily fluids, excreta, or DNA extracted from any of these).

### 14.4 Storage and Disposal of Information

Any study-related documentation aside from what is managed in “eACReSS” will be stored in a lockable filing cabinet, inaccessible to anyone other than the researchers in the Department of Cardiology. These materials will be kept for 10 years after study completion (person responsible for storage: the Principal Investigator). Other materials will be handled and retained appropriately according to the procedures of each participating medical institution.

Moreover, the information and data collected in this study will be stripped of personal identifiers (such as name, initials, patient ID) and replaced with a new code so that individuals cannot be identified, all under the responsibility of the Principal Investigator. After the storage period is over, any electronic data will be destroyed in a way that makes restoration impossible, and any paper documents will be shredded, paying attention to personal information. If each participating medical institution has separate procedures for disposal, those procedures will be followed.

**15. Payment of Money and Compensation**

### 15.1 Payment of Money (Participant’s Expenses)

In this study, the investigational device provided by Medtronic Japan Co., Ltd. will be used. Therefore, participants will not incur any costs for the investigational device (including ILR implantation and remote monitoring). The costs of tests and examinations conducted in this study are the same as those in standard medical care.

### 15.2 Compensation

If a participant in this study sustains health damage as a result of the study, the Principal Investigator, Sub-Investigators, and the participating medical institution will promptly ensure that the participant receives the appropriate diagnosis, treatment, and any other necessary measures. The Principal Investigator carries clinical research insurance with the coverage listed below, which will be applied in accordance with the insurance payment conditions to compensate for any losses suffered by the participant due to health damage resulting from the study (excluding cases where the participant is at fault):

- Compensation for death or permanent disability of the participant
- Medical expenses and medical allowances necessary for treating health damage of the participant

Additionally, the Principal Investigator and Sub-Investigators must be enrolled in medical malpractice liability insurance to cover potential health damages to participants arising from the normal scope of medical practice during this study.

**16. Disclosure of Information**

Before conducting this study, the required information set forth by the World Health Organization (WHO) to ensure the transparency of clinical research, as well as items that help the public make informed decisions about participating in clinical research, will be registered in jRCT and disclosed. The study will commence after disclosure in jRCT, and the information will be updated as needed depending on any changes to the implementation plan or progress of the study. Additionally, if a main outcome report or final study report is created, an outline of such a report will be made public.

**17. Study Period**

Overall study period: From the date the implementation plan is published on jRCT until September 30, 2030 (6 years)
(Planned enrollment period: 2 years; observation period: 3 years after enrollment ends; analysis period: 1 year)

**18. Explanation to Participants and Informed Consent**

### 18.1 Preparation of an Explanatory Document and Consent Form

To obtain consent from individuals participating in this study, a written explanation (the explanatory document) and a separate consent form must be created. In creating these documents, care should be taken to use plain language to aid participant understanding, and the following points must be included:

1. The title of this study, a statement indicating that its implementation has been approved by the administrator of the participating medical institution, and that the implementation plan has been submitted to the Minister of Health, Labour and Welfare
2. The name and title of the Principal Investigator
3. The reason why the participant has been selected for this study
4. The anticipated benefits and disadvantages of conducting this study
5. A statement that refusal to participate in this study is entirely voluntary
6. Information regarding withdrawal of consent
7. A statement that neither refusal to participate nor withdrawal of consent will result in disadvantageous treatment
8. How information related to this study will be disclosed
9. A statement that participants may request access or copies of the study protocol or other materials related to the conduct of this study, as well as how to make such a request
10. Information on protecting the participant’s personal information
11. The methods for storing and disposing of samples, etc.
12. The status of conflict-of-interest (COI) management in this study
13. The system for responding to complaints and inquiries
14. Cost-related matters for this study
15. Availability and details of alternative treatment methods, as well as a comparison of the anticipated benefits and disadvantages of those alternative methods
16. Matters concerning compensation and the provision of medical care for any health damage arising from this study
17. The name of the Certified Clinical Research Review Board that reviews this study, items reviewed by the board, and other matters related to the board
18. Other necessary matters related to conducting this study

The Principal Investigator will submit the explanatory document and consent form for approval by the Certified Clinical Research Review Board, and will then notify the Minister of Health, Labour and Welfare. Furthermore, if new information that could affect a participant’s willingness to continue in the study is obtained after consent is given, the explanatory document must be promptly revised and approved by the Certified Clinical Research Review Board, and then submitted to the Minister of Health, Labour and Welfare.

### 18.2 Obtaining Informed Consent

Using the explanatory document and consent form approved by the Certified Clinical Research Review Board and submitted to the Minister of Health, Labour and Welfare, the participant will be given sufficient explanation in writing and verbally without any coercion or undue influence. The participant will then sign and date the consent form to indicate free and informed consent. If information that may influence a participant’s decision to continue in the study (such as efficacy or safety updates) becomes available, or if changes in the implementation plan that may influence consent take place, the participant will be promptly informed, and their intention to continue participation will be confirmed in advance. Then, using the revised explanatory and consent documents—approved by the Certified Clinical Research Review Board and submitted to the Minister of Health, Labour and Welfare—re-consent will be obtained from the participant.

**19. Conflict of Interest**

This study will be conducted under a research contract with Medtronic Japan Co., Ltd., which will provide the investigational device (Medtronic LINQ II) and research funding. Other expenses required for this study will be covered by research funds (operational funds, etc.) of the Department of Cardiology. Statistical analysis will be performed by a statistician appointed by the Principal Investigator; Medtronic Japan Co., Ltd. will not be involved in the analysis.

All individuals involved in implementing this study must adhere to their respective institutions’ conflict-of-interest (COI) regulations regarding potential COIs related to the study’s implementation and results, and appropriately manage any such conflicts.

The Principal Investigator will verify the following points regarding COI in accordance with the COI management standards for this study and, after obtaining confirmation from the administrator(s) of the participating medical institution(s), will prepare a COI management plan and submit it to the Certified Clinical Research Review Board:

1. Any funding or other support from pharmaceutical or medical device companies for this study
2. Any donations, honoraria for writing or speaking engagements, or other contributions from pharmaceutical or medical device companies involved with the investigational product, provided to those who will conduct the study (Principal Investigator, Sub-Investigators, and statistical analysts) or to persons listed in the study protocol who would clearly benefit from conducting this study

Throughout the planning, execution, and reporting of this study, the Principal Investigator will continuously confirm whether any new COI situations that could influence the study results or their interpretation have arisen among those involved in the study, ensuring that the study does not infringe on the rights and interests of participants. If a new COI situation does arise, the Principal Investigator will revise the COI management plan and submit it to the Certified Clinical Research Review Board. Also, during the study period, at least once a year, the Principal Investigator will verify the COI status and report it to the Certified Clinical Research Review Board when submitting the regular study progress report.

**20. Intellectual Property Rights**

Any results or data obtained from this study will belong to the Principal Investigator (or the medical institution implementing the study). Usage rights will be determined in a separate contract.

Should any patent rights or other intellectual property rights arise (or be likely to arise) in connection with this study, the Principal Investigator will notify Medtronic Japan Co., Ltd., the manufacturer and marketer of the investigational device, and consult with them to decide on the ownership and handling of such rights.

**21. Handling of Personal Information**

### 21.1 Protection of Personal Information

When inputting personal information from the participating medical institution into the EDC system for this study, identifying information will be removed and replaced with a unique study participant identification code (Participant ID). Each institution will create and keep its own correspondence table to ensure the participant cannot be identified and will not provide this correspondence table to the EDC system. However, the monitor appointed by the Principal Investigator and the Clinical Research Review Board may inspect the records related to this study. In study reports, participants are identified only by the study-specific Participant ID, and their privacy must be protected when publishing the study results at conferences or in academic journals. Aside from the management procedures described in “14.4 Storage and Disposal of Information,” any separate rules and procedures concerning personal information protection established by the participating medical institution will be followed to ensure proper handling.

### 21.2 Secondary Use of Data

Data obtained in this study may be used again in the future after obtaining approval from the Certified Clinical Research Review Board or Ethics Committee, etc. If reused, the data may be kept longer than the period specified for retention of records. However, even in such cases, personal information will be protected by the same methods described in “21.1 Protection of Personal Information.”

**22. Adherence to and Changes in the Study Protocol**

### 22.1 Adherence to the Study Protocol

As long as the safety and rights of participants are not compromised, the Principal Investigator and Sub-Investigators will conduct this study in accordance with the study protocol.

### 22.2 Changes to the Study Protocol

1. If the Principal Investigator wishes to change the study protocol (including the explanatory document and consent form), the proposed changes must be reviewed by the Certified Clinical Research Review Board in advance. Afterward, the changes must be reported to the administrator of the participating medical institution according to the institution’s procedures. The Principal Investigator or Sub-Investigator must not conduct the study using the revised protocol (explanatory document and consent form) before obtaining approval from the Certified Clinical Research Review Board and the administrator of the institution.
2. If changes to the study protocol involve changes to the implementation plan, then after receiving approval from the Certified Clinical Research Review Board, the Principal Investigator must submit an amendment to the Minister of Health, Labour and Welfare. Neither the Principal Investigator nor any Sub-Investigator may conduct the study in accordance with the revised protocol (explanatory document and consent form) before the Minister of Health, Labour and Welfare has been notified of the amendment and it is published in jRCT.

**23. Managing Non-Compliance**

1. If the Principal Investigator becomes aware that this study is not being conducted in accordance with the Clinical Research Act, its enforcement regulations, or this study protocol (hereinafter “non-compliance”), they must promptly report it to the administrator of the participating medical institution. If a Sub-Investigator becomes aware of any non-compliance, they must promptly inform the Principal Investigator.
2. In cases of “serious non-compliance” (i.e., those that affect the rights or safety of clinical research participants, as well as the reliability of the study progress or results—examples include non-compliance with inclusion/exclusion criteria, discontinuation criteria, prohibited concomitant therapies, etc.), the Principal Investigator must promptly report the situation to the Certified Clinical Research Review Board. Furthermore, the administrator of the participating medical institution will publicly disclose the status of actions taken in response to such “serious non-compliance.”

**24. Periodic Reporting**

1. Each year, starting from the date the implementation plan for this study is published on jRCT, the Principal Investigator must, within two months of the end of that one-year period, report the following study implementation status to the administrator of the participating medical institution. The Principal Investigator will then submit the report to the Certified Clinical Research Review Board to hear their opinion on whether the study should continue.

(1) Number of participants enrolled in this study
(2) Occurrence of diseases or adverse events in this study and subsequent outcomes
(3) Occurrence of non-compliance and subsequent actions
(4) Evaluation of the safety and scientific validity of this study
(5) Matters related to conflict-of-interest management for this study

1. Within one month of the date on which the Certified Clinical Research Review Board gives its opinion, the Principal Investigator must report the following items to the Minister of Health, Labour and Welfare:

(1) The name of the Certified Clinical Research Review Board
(2) Whether the board deems it appropriate to continue this particular clinical research
(3) The number of participants enrolled in this study

**25. Discontinuation of the Study**

If the Principal Investigator decides to discontinue this study, the following measures will be taken:

1. Appropriate measures will be provided for participants in the study.
2. Within 10 days of the discontinuation date, the Principal Investigator will notify the Certified Clinical Research Review Board that the study has been discontinued, and also submit a notification to the Minister of Health, Labour and Welfare. If necessary, the board’s opinion will be obtained regarding the timing and method of ending the study or providing measures for the participants.
3. Even after notifying the Minister of Health, Labour and Welfare of the discontinuation, until the study is completed, reports of adverse events and periodic reporting must continue. Additionally, if any changes are made to the study progress that fall under changes to the implementation plan, an amendment must be submitted accordingly.

**26. Completion of the Study**

### 26.1 Preparation of the Final Report

When the period for collecting data on all evaluation items described in this protocol has ended, the Principal Investigator will, in principle, prepare a final report (a document summarizing the results of the clinical research) and its summary within one year of that end date. If the study is discontinued, the report must be prepared within one year of whichever is later: the discontinuation date or the date by which data collection was completed on all evaluation items.

The final report will include the following:

1. Background information on the participants (e.g., age, sex)
2. Information on study progress based on the study design (e.g., number of participants over time)
3. Summary of disease occurrence
4. Data analysis and results for the primary and secondary endpoints

If any changes have been made to the original statistical analysis plan, the details of those changes must be described in the final report.

### 26.2 Submission of the Final Report

1. Once the final report and its summary have been prepared, the Principal Investigator must seek the opinion of the Certified Clinical Research Review Board and promptly submit them to the administrator of the participating medical institution.
2. Within one month of the date on which the Certified Clinical Research Review Board provides its opinion on the final report and its summary, the Principal Investigator must submit the summary of the final report to the Minister of Health, Labour and Welfare, attaching the following documents:
   - The latest version of the study protocol (including the explanatory document and consent form)
3. When the final report summary is submitted to the Minister of Health, Labour and Welfare and published on jRCT, the Principal Investigator will inform the administrator of the participating medical institution.
