# Supplementary material for: Evaluation of continuous arrhythmia monitoring using an implantable loop recorder in heart failure patients with a reduced ejection fraction: The LINQ2-HF trial rationale and protocol
Source: PLoS One. 2025 Aug 28;20(8):e0321604. doi: 10.1371/journal.pone.0321604 (PMC12393711; doi:10.1371/journal.pone.0321604)
Supplement: Attachment — Submitted filename: Response to the reviewers.docx [file pone.0321604.s005.docx]

**Response to the editor and the reviewers’ comments**

We thank the editor and the reviewers for the insightful comments. We tried our best to address the issues raised by the editor and the reviewers, which helped us improve the quality of our manuscript significantly. We hope that the manuscript is now suitable for publication in PLOS One.

***Journal requirements:***

1. ***Please ensure that your manuscript meets PLOS ONE's style requirements,***

***including those for file naming. The PLOS ONE style templates can be found at*** [***https://journals.plos.org/plosone/s/file?id=wjVg/PLOSOne_formatting_sample_main_body.pdf***](https://journals.plos.org/plosone/s/file?id=wjVg/PLOSOne_formatting_sample_main_body.pdf)  ***and*** [***https://journals.plos.org/plosone/s/file?id=ba62/PLOSOne_formatting_sample_title_authors_affiliations.pdf***](https://journals.plos.org/plosone/s/file?id=ba62/PLOSOne_formatting_sample_title_authors_affiliations.pdf)

Thank you for your comment. We have revised the manuscript to comply with the PLOS ONE style guidelines, including font size and bold formatting. As the number of corrections is extensive, we have not listed each one individually; however, all changes have been highlighted for clarity.

2. ***Thank you for stating the following financial disclosure:***

***“Institute of Science Tokyo is the sponsor of the study with financial support from Medtronic Japan Co., Ltd (ERP-2023-13609).”***

***Please state what role the funders took in the study. If the funders had no role, please state: "The funders had no role in study design, data collection and analysis, decision to publish, or preparation of the manuscript." If this statement is not correct you must amend it as needed. Please include this amended Role of Funder statement in your cover letter; we will change the online submission form on your behalf.***

We thank your suggestion. We have added the following sentence to the Funding section as requested:
**Page 14, Line 247-248:** "The funders had no role in study design, data collection and analysis, decision to publish, or preparation of the manuscript."

3. ***Thank you for stating the following in your Competing Interests section:***

***“None declared.”***

***Please complete your Competing Interests on the online submission form to state any Competing Interests. If you have no competing interests, please state "The authors have declared that no competing interests exist.", as detailed online in our guide for authors at*** [***http://journals.plos.org/plosone/s/submit-now***](http://journals.plos.org/plosone/s/submit-now)

***This information should be included in your cover letter; we will change the online submission form on your behalf.***

Thank you for your guidance. Based on your instruction, we have included the following statement in the Competing Interests section:
**Page 14, Lines 230–231:** **"** *Drs. Miyazaki, Takigawa, and Goto are affiliated to a department that received an unrestricted research grant from Medtronic.*"

4. ***In the online submission form, you indicated that [The data are available from the***

***corresponding author upon reasonable request.].***

***All PLOS journals now require all data underlying the findings described in their manuscript to be freely available to other researchers, either 1. In a public repository, 2. Within the manuscript itself, or 3. Uploaded as supplementary information.***

***This policy applies to all data except where public deposition would breach compliance with the protocol approved by your research ethics board. If your data cannot be made publicly available for ethical or legal reasons (e.g., public availability would compromise patient privacy), please explain your reasons on resubmission and your exemption request will be escalated for approval.***

We appreciate your feedback. However, we would like to clarify that this is a study protocol paper, and therefore no data have been collected or are available at this time.

Therefore, the Data Availability Statement has been revised as follows:

**Page 14, Lines 254–255:** *“As this is a study design paper, no datasets were generated or analyzed during the current study.”*

5. ***Your ethics statement should only appear in the Methods section of your***

***manuscript. If your ethics statement is written in any section besides the Methods, please move it to the Methods section and delete it from any other section. Please ensure that your ethics statement is included in your manuscript, as the ethics statement entered into the online submission form will not be published alongside your manuscript.***

We appreciate your comment. We have moved the ethics statement to the Methods section in accordance with your instructions. The revised statement is now located as follows:
**Page 7, Lines 115–117:** "This study protocol was approved by the Institute of Science Tokyo Certified Review Board (NR2024-003). This study was conducted according to the guidelines of the Declaration of Helsinki."

In addition, the ethics statement was removed from the abstract and moved to the Methods section. The conclusion was revised accordingly.

**Page 2, Lines 40–42:** *“****Conclusion:*** *The LINQ2-HF trial is designed to detect new-onset AF and VT in HFrEF patients using ILR, with the aim of informing future strategies for arrhythmia management in this population.”*

***Reviewer #1:the manuscript is a study protocol that explores the use of an implantable loop recorder (ILR) for continuous arrhythmia monitoring in patients with heart failure with reduced ejection fraction (HFrEF).

1. The study is described as an exploratory pilot study with a sample size of 35 participants. why your sample size is 35?***

We appreciate the reviewer’s important comment. As per your suggestion, we have revised the statistical analysis section to clarify the rationale for our sample size. The following text has been added:

**Page 11, Lines 194–198:** “*In Japan, the use of ILRs in this study constitutes off-label use and is not covered by national health insurance. Therefore, device provision by Medtronic was necessary. Taking into account both the number of devices Medtronic could provide and the number of patients that could realistically be enrolled during the two-year recruitment period, the sample size was set at 35 participants for this exploratory study.*”

***2. Have you considered comparing ILR monitoring with historical data or previously published studies to strengthen the analysis?***

Thank you for your suggestion. We have addressed this point by citing previous studies that demonstrated ILR monitoring provides superior accuracy compared to traditional methods such as 24-hour ECG.
**Page 6, Lines 92–93:** “It has been shown that ILR enables up to 14% more accurate assessment compared to ECG during symptomatic episodes or 24-hour ECG monitoring.” ***3. The study must discuss and include ILR Sensitivity and Specificity.***

We appreciate your helpful feedback. As suggested, we have added information regarding ILR sensitivity and specificity in the manuscript.
**Page 6, Lines 91–92:** *“There is a previous study that demonstrates an ILR sensitivity of 95.2% and a specificity of 99.9%.”*

***Reviewer #2: The LINQ2-HF trial protocol presents a well-structured and clinically relevant study investigating the utility of ILRs for continuous arrhythmia monitoring in HFrEF. Given the strong association between undetected AF and ventricular arrhythmias with poor cardiovascular outcomes, this study has significant potential to contribute to heart failure management.
However, several issues need to be addressed to improve the study.

1. A comparison group using conventional ECG monitoring would provide stronger evidence of ILR effectiveness***

Thank you for this valuable comment. Our study is exploratory, and the sample size is relatively small; therefore, no comparative analysis is planned. Based on the findings of this study, we intend to conduct a subsequent study with a larger sample size, incorporating a formal sample size calculation to enable comparative analysis.

***2. Consider stratifying participants based on factors such as NYHA class, BNP/NT-proBNP levels, and prior heart failure hospitalizations to improve result interpretation.***

We thank the reviewer for the suggestion. BNP levels are assessed during the initial screening, and CHADS₂ score ≥1 is part of the inclusion criteria. Since the CHADS₂ score reflects several heart failure-related parameters, relevant clinical stratification will be inherently incorporated into the study through these criteria.

***3. It would also be valuable to assess and report lifestyle factors (e.g., diet, exercise, socioeconomic status) that may influence arrhythmia risk.***

We appreciate this important suggestion. Although lifestyle factors are not mandatory in our data collection, patients are followed at cardiology outpatient clinics, and relevant lifestyle information will be reviewed and noted from medical records whenever available.

***4. The protocol states that event rates will be estimated using Kaplan-Meier methods. While this is appropriate for time-to-event data, multivariable Cox regression analysis could further help adjust for potential confounders.***

Thank you for your comment regarding statistical methodology. We have incorporated the following sentence into the manuscript to reflect this addition:
**Page 11, Lines 200–201:** “To further explore factors associated with arrhythmia development, multivariable Cox regression analysis will be performed to identify predictors associated with the occurrence of AF and VT.”

***5. Will ILR findings be correlated with clinical outcomes such as hospitalization rates, stroke incidence, or mortality? Clarifying this would strengthen the study’s impact.***

Thank you for pointing this out. We apologize for any lack of clarity in the original description. As mentioned in the section on secondary endpoints, cardiovascular events will be assessed, including all-cause mortality, cardiovascular death, hospitalization due to heart failure exacerbation, and strokes. These outcomes will be analyzed in relation to ILR findings to evaluate their clinical significance.

***Reviewer #3: Interesting protocol. Some issues should be added

1. in the introduction it should be added benefit of this device when compared with intracardiac monitoring (some of these patients may need ICD)***

Thank you for this important perspective. As the reviewer rightly noted, some of the patients enrolled in this study may be candidates for ICD implantation. We have clarified this point by adding the following statement to the introduction.

**Page 6, Line 100-103: *“****Although some participants may be eligible for ICD implantation, certain individuals may be hesitant to proceed. In such cases, ILR findings may serve as objective evidence to support shared decision-making and promote more active engagement in treatment.****”***

***2. do you think that authors may exploit machine learning to improve detection of AF and also of monitoring of heart failure un these patients***

Thank you for your valuable comment. We would like to note that LINQ II incorporates the world’s first AI platform for ILRs, which applies artificial intelligence algorithms to data transmitted to the CareLink™ Network. This technology significantly reduces false alerts for atrial fibrillation and pauses, without compromising sensitivity.

For further details, please refer to: <https://europe.medtronic.com/xd-en/c/emea/cardiac-rhythm/linq-system.html#accurhythm>

***3. sample size calculation is needed***

We appreciate this important comment. As also raised by other reviewers, we have addressed this issue by adding the following description to the manuscript.

**Page 11, Lines 194–198:** “*In Japan, the use of ILRs in this study constitutes off-label use and is not covered by national health insurance. Therefore, device provision by Medtronic was necessary. Taking into account both the number of devices Medtronic could provide and the number of patients that could realistically be enrolled during the two-year recruitment period, the sample size was set at 35 participants for this exploratory study.*”

***4. it should be added who checked the loop recorder tracks***

Thank you for this pertinent comment. We have revised the manuscript to include the following clarification.

**Page 9, Lines 153–154:**“Data from the ILR remote monitoring system will be reviewed and validated by two board-certified cardiologists.”

***5. potential benefit should be more detailed***

We thank the reviewer for this valuable comment. Since this point partially overlaps with comment 1, we have incorporated additional explanation into the same section of the introduction. In addition, considering the potential benefits of early detection of atrial fibrillation, we have added the following sentence to the manuscript.

**Page 6, Lines 103-105:** *“Furthermore, early detection of AF may enable timely initiation of pharmacological therapy or catheter ablation before the onset of heart failure exacerbation or stroke.”*

We appreciate in advance for your excellent comments on this paper. We hope that our new version could correctly answer all the questions and is significantly improved over the previous submission.
