# Supplementary material for: Evaluation of continuous arrhythmia monitoring using an implantable loop recorder in heart failure patients with a reduced ejection fraction: The LINQ2-HF trial rationale and protocol
Source: PLoS One. 2025 Aug 28;20(8):e0321604. doi: 10.1371/journal.pone.0321604 (PMC12393711; doi:10.1371/journal.pone.0321604)
Supplement: Attachment — Submitted filename: Response_to_the_reviewers_auresp_2.docx [file pone.0321604.s006.docx]

**Response to the editor and the reviewers’ comments**

We thank the editor and the reviewers for the insightful comments. We tried our best to address the issues raised by the editor and the reviewers, which helped us improve the quality of our manuscript significantly. We hope that the manuscript is now suitable for publication in PLOS One.

***Reviewer #2:This study presents a clearly structured and clinically relevant study protocol addressing a significant gap in the management of HFrEF, the underdiagnosis of asymptomatic arrhythmias. The rationale is compelling, and the use of implantable loop recorders (ILRs) is timely given the limitations of conventional monitoring. The authors also demonstrate responsiveness to reviewer feedback, strengthening the manuscript substantially in the revision process. There are several issues that need to be addressed:***

***• The authors explained why only 35 patients were included, but they still need to add a statistical power calculation or effect size estimate to strengthen the study's reliability.***

Thank you for the suggestion. As previously noted, enrolling 35 consecutive patients represented the maximum feasible number at our center during the study period. Because our primary aim was to determine the real-world prevalence of AF and VT in patients with HFrEF—not to compare interventions or groups—we considered that a priori power or effect-size calculations—essential in comparative designs—were not applicable to this descriptive study. This is an exploratory study for future research.

***• Without a comparison group (like standard ECG or Holter), it's hard to judge how effective the ILR really is. Using historical or matched data could help.***

We appreciate the reviewer's important comment. We agree that an external benchmark is helpful. Reference 6 reports a 17 % prevalence of AF in HFrEF based on standard 12-lead ECG. We believe that this historical figure provides a useful point of comparison for the findings of our study.

***• Since this is a single-center study, the results may not apply widely. This limitation should be stated clearly.***

We appreciate your helpful feedback. As recommended, we have emphasized that the single-center nature of the study may restrict the generalizability of our findings as below.

**Page 12, Lines 227–229:*”****Although this was a single-center study and its findings may not be immediately generalizable, the results could still help shape clinical guidelines for ILRs in HF patients, potentially extending their use beyond current indications.****”***

***• The study mentions AI use in arrhythmia detection but doesn't explain how its accuracy will be measured. Future studies should assess how well the AI performs.***

We thank the reviewer’s insight. The ILR used in this study is the Reveal LINQ™ system, which employs the same algorithm evaluated in Reference 11. Because that publication provides detailed validation data, we have assumed that its validated performance also applies to our study population.

***• Lifestyle factors (like diet, exercise, smoking) were noted as important but aren't part of the formal data collection. Including them, even optionally, could provide useful insights.***

We appreciate this suggestion. Where information is available in the electronic medical record, we will now collect relevant lifestyle factors where available.

**Page 10, Lines 162–163:** “*Lifestyle factors, including diet and smoking history, will also be reviewed to the extent that information is available in the electronic medical record.*”

***• Define all abbreviations the first time they appear (e.g., NYHA is not explained in the text).***

We are very sorry for the lack of description of abbreviation. All abbreviations have now been defined at their first appearance in the manuscript.

**Page 5, Lines 74–76:** *“Typically, a low ejection fraction combined with HF symptoms (New York Heart Association (NYHA) functional class Ⅱ-Ⅲ) is a key criterion for an implantable cardioverter defibrillator (ICD) implantation as primary prevention.”*

We appreciate in advance for your excellent comments on this paper. We hope that our new version could correctly answer all the questions and is significantly improved over the previous submission.

***Reviewer #3: All comments have been addressed and authors should be complimented for performing such an accurate rebuttal. I think it is worth of publication.***

We sincerely thank the reviewer for the encouraging feedback and for acknowledging how carefully we addressed every comment. We greatly appreciate the recommendation for publication.
